# Wooden breast myopathy is characterized by satellite cell dysfunction and syndecan-4 shedding

**DOI:** 10.3389/fphys.2024.1513311

**Published:** 2024-12-23

**Authors:** Lucie Pejšková, Addolorata Pisconti, Marianne Lunde, Ka Yi Ho, Nina Therese Solberg, Shiori Koga, Erik Tengstrand, Cathrine Rein Carlson, Mona Elisabeth Pedersen, Sissel Beate Rønning

**Affiliations:** ^1^ Raw Materials and Optimalization, Nofima AS, Ås, Norway; ^2^ Department of Biochemistry and Cell Biology, SUNY Stony Brook, Stony Brook, NY, United States; ^3^ Institute for Experimental Medical Research, Oslo University Hospital and University of Oslo, Oslo, Norway

**Keywords:** wooden breast, syndecan-4, myopathy, syndecans, broiler chicken, skeletal muscle satellite cells

## Abstract

**Introduction:**

Skeletal muscle satellite cells (MuSCs or stem cells) play a crucial role in muscle development, maintenance, and regeneration, supporting both hypertrophy and regenerative myogenesis. Syndecans (SDCs) act as communication bridges within the muscle microenvironment, regulating interactions with extracellular matrix components and contributing significantly to tissue repair and inflammation. Specifically, syndecan-4 (SDC4) is involved in muscle regeneration at multiple stages.

**Methods:**

This study delves into the emerging challenge of wooden breast (WB) myopathy and its connection with SDC4. Our hypothesis proposes that disruptions in MuSC dynamics through SDC4 contribute to the increased incidence of breast myopathies observed in growing broilers. To test our hypothesis, non-affected and affected broilers were systematically selected, and the characteristics of WB myopathy were studied both *in vitro* and *in vivo*. SDC4 overexpression in MuSCs and blocking peptides (BPs) corresponding to the SDC4 ectodomain were used for investigating the role of SDC4 in muscle development and its shedding levels.

**Results and discussion:**

*In vivo* examination of affected muscles revealed smaller fibers and changes in metabolic pathways. *In vitro* studies unveiled disrupted proliferation of MuSCs in WB myopathy, accompanied by the downregulation of several muscle markers. Investigation of the potential role of SDC4 in the pathogenesis of WB myopathy revealed a decreased tendency in SDC4 gene expression and increased shedding of its ectodomain. Moreover, we showed that SDC4 overexpression is linked to reduced proliferation in MuSCs and affected myogenesis. We detected an impaired proliferation of WB-affected MuSCs, revealing critical insights into the dysfunctional state of these cells in myopathy. Additionally, by treating MuSCs with blocking peptides derived from the SDC4 ectodomain, we identified altered proliferation. Taken together, this work contributes with valuable knowledge on the molecular mechanisms underlying WB myopathy and the role of SDC4 in this chicken myopathy.

## 1 Introduction

Commercial broiler chickens have undergone genetic selection for effective growth and higher breast muscle yield in response to increasing demand for poultry meat (Petracci and Cavani, 2012; Zuidhof et al., 2014; Petracci et al., 2015). However, this intensified selective breeding has led to an increase in production-related diseases, particularly wooden breast (WB) myopathy (Sihvo et al., 2014; Petracci et al., 2019). This condition predominantly affects the *pectoralis major* muscle in broiler chickens. WB myopathy results in firm, woody-like breast muscles, negatively affecting meat quality, animal welfare, and economic viability (Sihvo et al., 2014; Mudalal et al., 2015; Velleman, 2015; Velleman et al., 2019; Sanden et al., 2021).

Wooden breast is characterized by skeletal muscle fibrosis, necrosis, and multifocal degeneration of muscle tissue previously demonstrated both by us (Wold et al., 2017; Sanden et al., 2021; Pejšková et al., 2023) and others (Sihvo et al., 2014; Velleman and Clark, 2015; Soglia et al., 2016). Furthermore, the infiltration of inflammatory cells, muscle fiber myodegeneration with partial regeneration, reduced angiogenesis, and adipose infiltrations have also been demonstrated (Mutryn et al., 2015; Papah et al., 2017; Hosotani et al., 2020). The etiology of WB is still not completely identified, but hypoxia and oxidative stress are suggested as two major contributors to its onset (Hosotani et al., 2020; Bordini et al., 2024). Other mechanisms relevant to the onset and progression of WB include dysregulation of energy metabolism, mitochondrial, dysfunction, and a profound change in extracellular matrix composition (Hosotani et al., 2020; Young and Rasmussen, 2020).

Skeletal muscle satellite cells (MuSCs), also known as muscle stem cells, located between the basement membrane and the myofiber plasma membrane, are the primary contributors to postnatal growth, maintenance, and skeletal muscle regeneration (Ganassi et al., 2022; Careccia et al., 2024). MuSCs remain quiescent under normal conditions, interacting closely with myofibers and potentially with endothelial cells (Christov et al., 2007; Chakkalakal et al., 2012; Yin et al., 2013). In response to various stimuli and injuries, MuSCs become activated, proliferate, and differentiate, thus contributing to muscle tissue formation (Motohashi and Asakura, 2014; Mashinchian et al., 2018). The typical process of muscle fiber regeneration involves several sequential steps: initial inflammation accompanied by the removal of necrotic tissue, subsequent angiogenesis to re-establish blood flow, activation of MuSCs, migration and proliferation to the injured area, maturation of myotubes, and eventually innervation, leading to the formation of fully functional new muscle fibers (Tidball, 2011).

Myogenesis is regulated by a series of transcription factors, including Paired box 7 (*PAX7*), which is expressed in quiescent, activated, and proliferating MuSCs; myogenic factor 5 (*MYF5*), whose expression overlaps with PAX7 at the mRNA levels; and myogenic determination protein 1 homolog (*MYOD1*), which is expressed immediately after activation and continues through myotube formation (Zammit et al., 2006; Careccia et al., 2024). Once the cells progress through differentiation, other myogenic transcription factors, such as myogenin (*MYOG*) and different isoforms of skeletal muscle-specific myosin heavy chain 1B (*MyH1B*), are upregulated (Olguín and Pisconti, 2012; Careccia et al., 2024).

Regenerative mechanisms have been suggested to be impaired in chickens affected by WB syndrome (Velleman, 2015; Velleman, 2023), and modern commercial chickens experience decreased regeneration of damaged myofibers due to reduced MuSC myogenic activity in the *pectoralis major* muscle (Xu and Velleman, 2023). We have recently shown that the affected WB phenotype is characterized by more than 4,000 upregulated genes compared with the non-affected phenotype, as well as extensive extracellular matrix (ECM) remodeling, increased matrix metalloproteinase (MMP) activity, and altered expression and shedding levels of the syndecan protein family of four members (SDC1–4) (Pejšková et al., 2023).

SDCs are type I transmembrane proteins modified with glycosaminoglycan (GAG) chains, predominantly heparan sulfate, which play a pivotal role in mediating cell–cell and cell–matrix interactions (Couchman, 2010). Their cytoplasmic domains activate signaling pathways that regulate various cellular and transcriptional functions. As co-receptors, SDCs collaborate with integrins, growth factors, and other signaling molecules, influencing diverse biological processes such as cell adhesion, proliferation, differentiation, and ECM assembly (Afratis et al., 2017).

Moreover, SDCs are regulators of skeletal muscle growth and tissue repair (Pisconti et al., 2012; Rønning et al., 2015; Pisconti et al., 2016; Rønning et al., 2020; Jones et al., 2022; Sztretye et al., 2023), inflammation, and tumor progression, emphasizing their multifaceted roles in cellular processes (Xian et al., 2009; Gopal, 2020). All SDCs are expressed in MuSCs, and it has been shown that the activation of MuSCs occurs in an SDC-dependent manner in mice (Pisconti et al., 2012; Pisconti et al., 2016; De Micheli et al., 2020). This finding is also in accordance with studies in chickens addressing SDC4 (Velleman and Song, 2017). In the context of WB, a noteworthy aspect of SDCs lies in their regulatory mechanisms, involving the shedding of their ectodomains through various sheddases, including MMP2 and MMP9 (Manon-Jensen et al., 2013; Pejšková et al., 2023). We have recently identified the shedding of SDCs and increased SDC4 gene expression *in vivo* to be involved in WB pathogenesis (Pejšková et al., 2023).

The present study aimed to characterize metabolic changes and muscle fibers in WB-affected chickens compared with non-affected chickens. Furthermore, we investigated the proliferation and differentiation potential of MuSCs isolated from normal and WB-affected chickens and their connection with SDC4 and its shedding.

## 2 Materials and methods

### 2.1 Animal handling and sampling

A total of 60 samples of breast muscle *(Pectoralis major)* were acquired from male Ross 308 broiler chickens (*Gallus Gallus*) at 36 days post-hatching (Pejšková et al., 2023). These chickens were raised with *ad libitum* access to a pelleted wheat/maize diet from the 10th day of age, housed in pens of dimensions 2.4 × 0.95 m with wood shavings, exposed to a lighting regimen of 6 h of light and 18 h of darkness, and subjected to a gradually decreasing temperature from 28°C to 21°C. The sampling procedure involved initial palpation-based sorting, followed by classification using histological methods and near-infrared (NIR) spectroscopy in combination with RNA-seq analysis. In our previous investigation (Pejšková et al., 2023), the samples showed a clear separation between birds with pronounced signs of fibrosis and collagen infiltration (hereafter termed “affected”) those with fewer signs (hereafter termed “non-affected”).

Muscle tissue specimens were obtained post-mortem from the breed chickens at NMBU, Norway. Given their conformity to established regulatory norms in food production, obtaining approval from the Regional Ethics Committee (REC) or the Norwegian Centre for Research Data (NSD) was deemed unnecessary. In accordance with Norwegian legislation governing the experimental use of animals (FOR-2015-06-18-761 §2a), ethical clearance is not obligatory for sample collection from animals slaughtered or utilized in non-experimental agricultural and aquacultural activities. This exemption was corroborated through direct correspondence with the Norwegian food safety authority, Mattilsynet.

### 2.2 *In vivo* tissue sample preparation and *in vitro* satellite cell isolation

*In vivo* tissue samples were prepared as described previously by Pejšková et al., 2023. Samples for RT-qPCR, RNA-seq, and proteomics were collected immediately after slaughter, snap-frozen in liquid nitrogen, and stored at −80°C until further analysis. For the microscopy immunofluorescence analysis, pieces of approximately 8 mm × 8 mm × 2 mm were cut from the outer layer in the upper part of the *pectoralis major* for all the animals after slaughtering, fixed in IHC Zinc Fixative (#550523, BD Pharmingen, New Jersey, USA) for 24 h before dehydrating and paraffin embedding. All animals were first sorted by palpation and then classified by histology and NIR spectroscopy (Pejšková et al., 2023) (hereafter termed NA (non-affected) and A (affected)).

Chicken primary muscle satellite cells (hereafter termed MuSCs) were isolated from biopsies collected from breast muscle (*pectoralis major*) obtained from the chicken sample groups described above or from breast fillet samples with or without WB from Ross 308 of the same age collected at the industrial abattoir and classified as non-affected or affected by NIR and histology (Nortura AS, Hærland, Norway). Tissue samples (2–5 g) were kept in PBS with 0.5% penicillin/streptomycin (10,000 units/mL) and 0.5% Fungizone before being minced using a knife on Petri dishes, and the resulting minced pieces of meat were transferred to tubes containing 10 mL collagenase (#C2674, Sigma-Aldrich) solution, followed by a 1-h incubation at 37°C with agitation at 70 rpm in a water bath. After a brief centrifugation at 550 RCF for 10 s, the supernatant was filtered using a 100-μm strainer and transferred to a new tube containing 10% fetal bovine serum (FBS). The tissue was further digested using 0.05% trypsin/EDTA for 1 h/70 rpm in a 37°C water bath, followed by 10% FBS addition for enzyme inactivation. The cell suspension was centrifuged, and the supernatant was again filtered using the strainer. Cells from both pellets were pooled and re-suspended in 3 mL of cell growth medium (DMEM 1X (#41965-047, Thermo Fisher Scientific, MA, USA), 20% chicken serum, 2% chicken embryo extract (#ICNA092850145, MP Biomedicals, CA, United States), 2% Ultroser™ G serum substitute (#15950-017, Sartorius AG, Germany), 0.5% penicillin/streptomycin (10,000 units/mL), and 0.5% Fungizone) and plated on uncoated 25-cm^2^ flasks. For the removal of the fast-adhering fibroblast cells from the primary muscle cells, the cells were placed in uncoated cell flasks for 1 h at 37°C. The non-adhering MuSCs were then collected and further seeded in 25-cm^2^ flasks coated with 3 μL/cm^2^ entactin-collagen IV-laminin (ECL, 1 mg/mL, Millipore, Billerica, MA, USA). Cell culture media was changed every 48 h during the cell culture process. The isolated cells were proliferated, split into 75 cm^2^ or 175 cm^2^ coated culture flasks, and then stored in liquid nitrogen in a growth medium containing DMSO until further use. All experiments were performed at the 3rd, 4th, or 5th passage.

Following the isolation of primary chicken cell lines, the growth medium was transitioned from DMEM 1x supplemented with 20% chicken serum, 2% chicken embryo extract, 2% Ultroser™ G serum substitute, 0.5% penicillin/streptomycin (10,000 units/mL), and 0.5% Fungizone to DMEM 1x containing 10% FBS, 2% Ultroser™ G serum substitute, 0.5% penicillin/streptomycin (10,000 units/mL), and 0.5% Fungizone for experimental purposes. Cell counting was performed using the automated cell counter NucleoCounter^®^ NC-202™ (ChemoMetec A/S, Lillerød, Denmark). The cells were seeded on ECL-coated 96-well plates with a concentration of 2000 cells/well for the proliferation assay and 25,000 cells/well for the differentiation assay. Using Incucyte SX3/SX1 (Sartorius AG, Germany), the cells were monitored for 7 days in the case of proliferation in the incubator at 37°C and 5% CO_2_. The differentiation experiment was induced by a complete medium without FBS serum and Ultroser G in 85%–100% confluence after 1 day of proliferation and monitored for another 2 days.

### 2.3 Inhibition and transfection of MuSCs

MuSCs from both affected and non-affected groups (n = 3 each) were seeded at a density of 2000 cells per well in 96-well plates or 48,000 cells per well in 6-well plates. For the inhibition experiments, cells at 50% confluence were treated with inhibitors targeting the MEK/ERK pathway (#PD0325901, N-[(2R)-2,3-dihydroxypropoxy]-3,4-difluoro-2-[(2-fluoro-4-iodophenyl) amino]-benzamide, STEMCELL Technologies) and p38 MAPK pathway (#SB202190, 4-[4-(4-fluorophenyl)-5-(4-pyridinyl)-1H-imidazol-2-yl]-phenol, STEMCELL Technologies). Each inhibitor was dissolved in DMSO and administered to the well to reach a concentration of 25 µM.

MuSCs were transfected with chicken SDC4 (in pCEP4, UniProt #P49416, custom-made by GenScript Corp.) at 50% confluence using the Lipofectamine 3000 Transfection Kit (#L3000-008, Thermo Fisher Scientific) following the manufacturer’s protocol. To assess transfection efficiency, pEGFP-N1 (GenScript Corp.) was used as a readout, with 2.5 μg of DNA used per well. MuSCs were transfected for 24 h before lysis or treatment with blocking peptides. Blocking peptide (BP) sequences, corresponding to the ectodomain of chicken SDC4 protein (custom-made by GenScript Corp.), with underlined overlapping sequences and different solvents are provided in Table 1. BPs were used at a concentration of 2.5 µM for 2 h during the treatment of proliferating MuSCs.

**TABLE 1:** Custom-made blocking peptides and their solvents.

Name	Sequence	Solvent
BP1	MPLPRAAFLLGLLLAAAAAESVRETETMDA	3% NH_3_
BP2	ETETMDARWLDNVGSGDLPDDEDIGEFTPH	H_2_O
BP3	FTPHLTSDEFDIDDTSGSGDYSDY	DMSO
BP4	YSDYDDAIYLTTVDTPAISDNYIPGDTERK	H_2_O
BP5	PGDTERKMEGEKKNTMLDNEIIPDKASPVE	H_2_O

### 2.4 Reverse transcription-quantitative PCR and RNA sequencing

RT-qPCR: the total RNA for RT-qPCR was isolated from tissue lysate (n = 8 in each group) or proliferating MuSCs (n = 8 in each group) using the RNeasy Mini Kit (#74104, QIAGEN, Germany) according to the manufacturer’s instructions.

The total RNA from tissue was prepared from approximately 100 mg of tissue by homogenization in the RLT buffer containing 2 mM DTT using the Precellys Lysing Kit (#P000911-LYSKO-A.0, Bertin Technologies, France), 4 × 20 at 6000 rpm with 10 break between shakes, followed by a 10-min spin at 5000 g. Samples were incubated with Proteinase K (#19131, QIAGEN, Germany) according to the manufacturer’s instructions. cDNA was generated from 2 µg of total RNA using TaqMan Reverse Transcription Reagents (#N8080234, Thermo Fisher Scientific, MA, United States) in a 40 µL reaction volume with random hexamers according to the manufacturer’s protocol.

Proliferating MuSCs (n = 8 in each group) were seeded in a 6-well plate at 12,000 cells/well and allowed to proliferate for 3 days; after washing twice, 350 µL of the RLT buffer containing 2 mM DTT was added. cDNA was generated from 290 ng of total RNA using the LunaScript RT SuperMix Kit (#E3010L, New England Biolabs, MA, USA) in a 30 µL reaction volume with random hexamers according to the manufacturer’s protocol. RT-qPCR analysis was carried out using the Luna Universal probe RT-qPCR Master Mix (#M3004X, New England Biolabs, MA, USA) and QuantStudio 5 (Applied Biosystems, Foster City, CA, USA) PCR System. The amplification protocol was initiated at 95°C for 1 min by initial denaturation, followed 40 cycles of denaturation at 95°C for 15 s and then extension at 60°C for 30 s.

RT-qPCR analyses were performed with three technical replicates from each sample. The relative gene expression was calculated using the comparative 2^−ΔCt^ (Schmittgen and Livak, 2008; Bustin et al., 2010) method for the tissue and cell culture experiments comparing non-affected vs. affected samples. Non-affected MuSCs transfected with SDC4 were additionally analyzed and compared to the control using the 2^−ΔΔCt^ method. In short, the values are generated by subtracting reference gene EEF2 values for each sample to obtain ΔCt values, and for ΔΔCt values, the values were related to the average gene expression of the untransfected control (Lipofectamine, MuSCs) for each gene. The relative gene expression is then calculated using formula 2-^ΔCt^ or 2^−ΔΔCt^ in the case of non-affected MuSC transfection. All TaqMan^®^ primers and probes are listed in Table 2.

**TABLE 2:** Gene target and TaqMan^®^ primer/probe assays.

Gene target	TaqMan®primer/probe assays
*EEF2*	Gg03339740_m1
*Mki67*	Gg07186598_s1
*CDKN2A*	Gg07157676_m1
*CDKN1A*	Gg03814244_s1
*MYOG*	Gg03363788_m1
*MYOD1*	Gg03363970_m1
*SDC1*	Gg07175697_s1
*SDC2*	Gg03345644_m1
*SDC3*	Gg03339851_m1
*SDC4*	Gg03370419_m1
*PAX7*	Gg03348488_m1

RNAseq: total RNA was extracted from muscle tissue samples stored at −80°C using the RNAdvance Tissue Kit (Beckman Coulter, IN, USA) following the manufacturer’s guidelines. A NanoDrop Spectrophotometer (Thermo Fisher Scientific, MA, United States) was utilized to assess RNA purity and concentration of all samples, while the integrity of nine randomly selected samples was evaluated using a 4150 TapeStation RNA Screen Tape (Agilent, CA, United States). Extracted RNA (1.5–4.5 µg) was subsequently dispatched to a commercial sequencing provider (Novogene, United Kingdom), where quality reassessment confirmed the lowest RNA Integrity Number (RIN) to be 9.1. Sequencing libraries were prepared using NEBNext Ultra Directional RNA Library Prep Kits (NEB, MA, United States) and subjected to PE150 sequencing on NovoSeq 6000 instruments with S4 flow cells (Illumina, CA, United States). Quality control of reads was performed using FastQC (Andrews, 2010), and raw reads underwent trimming with fastp (Chen et al., 2018) to eliminate adapter sequences. Gene expression levels were determined by quantifying transcript expression with Salmon (Patro et al., 2017) using the GRCg7b reference genome from NCBI, and the data were summarized at the gene level. DESeq2 (Love et al., 2014) facilitated the analysis of differentially expressed genes between affected and normal groups. The R package clusterProfiler (Yu et al., 2012; Wu et al., 2021) was employed to estimate Gene Ontology (GO) and Kyoto Encyclopedia of Genes and Genomes (KEGG) pathway enrichment for upregulated and downregulated gene sets separately.

For RNAseq of MuSCs (n = 19), RNA was extracted using the RNeasy Mini Kit and sent to Novogene for library preparation and sequencing. At Novogene, messenger RNA was purified from total RNA using poly-T oligo-attached magnetic beads. After fragmentation, the first-strand cDNA was synthesized using random hexamer primers, followed by the second-strand cDNA synthesis. The library was ready after the end repair, A-tailing, adapter ligation, size selection, amplification, and purification. The library was quantified using Qubit and real-time PCR, and size distribution was detected using a bioanalyzer . The quantified libraries were pooled and sequenced on an Illumina NovoSeq 6000 instrument. Sequencing QC was performed using FastQC v0.12.1 (Andrews, 2023), Trim Galore v0.6.7 (Krueger et al., 2021), and Cutadapt v3.4 (Martin, 2011). Reads were mapped to the GRCg7b version of the *Gallus* reference genome using STAR v2.7.9a (Dobin et al., 2013). Alignments were converted to BAM format and sorted using SAMtools v1.17 (Li et al., 2009). Transcript expression was then quantified with Salmon v1.10.1 (Patro et al., 2017) and converted to gene-level counts with Tximport v1.12.0 (Soneson et al., 2015). Differential expression analysis comparing the affected and not affected groups was next performed using the nf-core differential abundance pipeline v1.4.0 (Cristina Tuñí i Domínguez et al., 2023), with read count normalization and statistical analysis performed using DESeq2 v1.34.0 (Love et al., 2014). Initial exploratory analysis showed that two samples from the “not affected” group were statistical outliers based on the median absolute deviation of their normalized read counts, and these samples were excluded from further analysis. This left nine remaining samples in the not affected group and 10 samples in the affected group. The results of the differential expression analysis were visualized using the Enhanced Volcano R package v1.20.0 (Blighe and Lun, 2018).

Gene Set Enrichment Analysis (GSEA) was performed with the ClusterProfiler R package v4.10.1 (Yu et al., 2012; Wu et al., 2021) using the FGSEA algorithm (Korotkevich et al., 2016). Log2 fold-change scores were used to rank genes, and the results were visualized using the enrichplot package v1.22.0 (Wu et al., 2021). Network analysis was performed to find genes with correlated expression levels with WGCNA v.1.72.5 (Langfelder and Horvath, 2008) using normalized and variance-stabilized read counts and a soft-thresholding power value of 10. The genes present in selected modules were then subject to functional overrepresentation analysis (ORA) using ClusterProfiler v4.10.1. For the ORA, the background gene list was defined as the set of genes used in the differential expression analysis that also possessed relevant functional annotations.

### 2.5 Western blotting

Proliferating MuSCs were seeded at a concentration of 30,000 cells per well in a 6-well plate and allowed to proliferate for 3 days; after washing twice with PBS, the cells were lysed with 100 µL of RIPA buffer containing a phosphatase inhibitor cocktail 2 (#P5726, Sigma-Aldrich, Merck) and AEBSF protease inhibitor (#78431, Thermo Scientific, United States) for 30 min on ice. Samples were centrifuged at 13.000× g for 30 min at 4°C. The supernatant, containing soluble proteins, was collected and stored at −80°C until analysis. The protein concentration was determined using the Micro BCA Protein Assay Kit (#PIER23235, Thermo Fisher Scientific, Waltham, MA, USA).

Samples were prepared by mixing with 4x sample buffer, consisting of 5 g sucrose (#16104, Sigma Merck, Darmstadt, Germany), 3.75 mL 20% SDS (L3771, Sigma Merck), 1.25 mL 0.5 M Tris-HCl (pH 6.8) (T5941, Sigma Merck), 310 mg DTT (D9779, Sigma Merck), 1 mL of 0.1% bromophenol blue (B5525, Sigma Merck), and 10 mL of MQ H_2_O and boiling for 5 min at 95°C. In addition, 10 μg/μL of proteins were loaded onto a 4%–15% Criterion TGX Precast Gel (#5671084, Bio-Rad, Hercules, CA, United States). Precision Plus Protein All Blue Standards (#1610373, Bio-Rad) and Precision Plus Protein Dual Color Standards (#1610374, Bio-Rad) were used as standard molecular weights. The gels were blotted onto PVDF Western blotting membranes (#03010040001, Sigma Merck) with extra thick blot filter paper, precut (#1703967, Bio-Rad), using the Trans-Blot Turbo system (Bio-Rad). After transfer to membranes, the membranes were rinsed in ddH_2_O, and the water was discarded. The membranes were then incubated in 10 mL of Revert 700 Total Protein Stain solution (cat# 926-11021, LI-COR, Lincoln, NE, United States) for 5 min with gentle shaking. The Total Protein Stain Solution was then decanted completely, and the membranes were rinsed two times for 30 s with 10 mL of wash solution (6.7% glacial acetic acid and 30% methanol in water). The membranes were imaged immediately in the 700 nm (IR700) channel with Azure 600 (Azure Biosystems, Redmond, WA, United States). After imaging, the membranes were washed for 15 min in 1x TBS-T to remove the turquoise color from the stain. The PVDF membranes were blocked in 1% casein (Western Blocking Reagent, #11921681001, Sigma Merck) and TBS-T for 60 min at room temperature (RT), followed by incubation with primary antibodies such as SDC1–4 (1:1,000, custom-made, GenScript, Piscataway, NN, United States), p38 (M138) (1:1,000, #ab31828, Abcam, United Kingdom), Phospho-p38 MAPK(Thr180/Tyr182) (1:1,000, #9211, Cell Signaling, MA, USA), p44/42 MAPK (Erk1/2) (1:1,000, #4695, Cell Signaling), or Phospho-p44/42 MAPK (Erk1/2) (Thr202/Tyr204) (1:1,000, #9101, Cell Signaling) overnight at 4°C. Membranes were washed three times for 10 min each in TBS-T before being incubated with horseradish-peroxidase-conjugated secondary antibodies (anti-mouse IgG, NA931V, or anti-rabbit, NA934V, both from Cytiva, Marlborough, MA, USA) diluted 1:3,000 in TBS-T, goat anti-rabbit IgG and goat anti-mouse IgG secondary antibodies, HRP-conjugated (1:5000, #31460, #31430, Invitrogen), followed by three additional times 10-min washes in TBS-T. Blots were developed using ECL Prime (#RPN2236, GE Healthcare, IL, United States) or SuperSignal West Pico PLUS Chemiluminescent Substrate (#34577, Thermo Fisher Scientific, MA, United States) for chemiluminescence signal detection. The chemiluminescence signals were detected using Azure 600 (CA, United States) or iBright™ CL1500 Imaging System (Invitrogen). The membranes were reprobed with reference protein anti-GAPDH (#sc-47724, Santa Cruz, CA, United States) after stripping using the Restore Western blot Stripping buffer for 5 min at RT (#21063, Thermo Fisher Scientific, MA, United States) and washed 3 × 15 min in TBS-T before blocking. Quantification of Western bands was conducted using ImageQuant 10.2.499 (Cytiva).

### 2.6 Label-free quantitative mass spectrometry-based proteomics

Twelve biological replicates of each group, affected and non-affected samples, were included in the analysis. Salt-soluble proteins were extracted from approximately 100 mg chicken breast fillet using 1,000 µL of extraction buffer [10 mM Tris, pH 7.6; 1 mM EDTA; 0.25 M sucrose]. Samples were homogenized twice for 20 s at 6,000 rpm speed with a 5s pause between the homogenization steps using Precellys^®^24 (Bertin Technologies) and centrifuged at 16000 *g* for 15 min. The protein concentration was determined using the RC DC™ Protein Assay (#5000121, Bio-Rad Laboratories, Inc, United States). A measure of 60 µg of proteins was reduced with 0.1 M DTT, alkylated with 55 mM 2-iodoacetamide, and digested with trypsin/Lys-C (#V5071, Promega, United States) at a 1:30 (w/w) enzyme-to-protein ratio on a Microcon-10 YM (Merck Millipore, United States) centrifugal filter unit at 37°C overnight. The peptide concentration was measured using the NanoDrop One at 205 nm, and 10 µg of peptides were purified and concentrated using a StageTip following the protocols (Rappsilber et al., 2007; Yu et al., 2014). Peptides were resolved with a loading buffer [2% (v/v) ACN; 0.05% (v/v) trifluoroacetic acid], and 1 µg of peptides was analyzed using a nano-UHPLC coupled with a Q Exactive Quadrupole-Orbitrap Mass Spectrometer (Thermo Fisher Scientific, United States) at the MS/Proteomics Core Facility at Campus Ås. Detailed liquid chromatography-tandem mass spectrometry (LC-MS/MS) settings can be found in Koga et al. (2019). Peptides from the 12 most intense peaks obtained during the 120-min elution were fragmented, and the mass-to-charge ratios of these fragmented ions were measured (MS/MS). Mass spectral data were processed using MaxQuant version 2.3.1.0 (Cox and Mann, 2008). The parameters for protein identification included trypsin/Lys-C specificity with reference to the proteome database for broiler chicken, *Gallus gallus* (Entry nr. UP000000539), downloaded from UniProt (43710 entries). Other parameters were maintained as defaults, and the label-free quantification (LFQ) algorithm was employed for protein quantification. LFQ intensity values from MaxQuant were imported into Perseus software version 2.0.6.0 (Tyanova et al., 2016) for statistical analysis and visualization of the data. Prior to analysis, proteins identified only by site, reverse sequence, and potential contaminant proteins were excluded. LFQ intensities were log2-transformed, and proteins possessing valid LFQ intensity values in more than 70% of biological replicates for at least one of the groups (non-affected and affected) were retained for further analyses. Welch’s T-tests, with a 95% confidence limit taking the false discovery rate (FDR) into account, were carried out for all identified proteins between non-affected and affected chicken groups. GO enrichment analyses were carried out for the significantly differentially expressed proteins with g:GOSt on the online g:Profiler platform (Kolberg et al., 2023) with the following data sources: GO terms biological processes (GO: BP), KEGG, and Human Phenotype Ontology (HPO). Gene names for the four proteins were unknown, and therefore, they were omitted from the GO enrichment analysis. Ensemble IDs with the most GO annotations were selected for three proteins (gene names: *RBP4*, *TTR*, and *ACTG1*). Moreover, missing values for LFQ intensities were imputed from a normal distribution (default setting of width 0.3 and downshift 1.8) in Perseus, and principal component analyses were carried out using MATLAB 2022b (The MathWorks, Inc, Natick, MA, USA).

### 2.7 Immunohistochemistry and immunofluorescence

The histology scoring of the samples used in this research was performed by staining with hematoxylin and eosin (H&E) according to standard procedures and previously described in Pejšková et al., 2023. To perform immunofluorescence analyses, tissue sections of affected and non-affected WB samples (N = 8–11 for each group) were deparaffinized, rehydrated with xylene, followed by gradually decreasing ethanol concentration (100%, 95%, 70%, and 50%) and ddH_2_O (Milli-Q), and then washed once with PBS pH 7.4 for 3 min. Sections were permeabilized at RT with 0.5% Triton X-100 (#X100, Sigma-Aldrich, Merck) in PBS (PBS-T) for 10 min and then washed twice in PBS-T pH 7.4 for 5 min each. For muscle morphometry, sections were incubated with DAPI (4′,6-diamidino-2-phenylindole dihydrochloride) (#D1306, 2 mg/mL, Invitrogen, MA, USA), wheat germ agglutinin, and Alexa Fluor™ 555 Conjugated (#W32464, 1:1000, Thermo Fisher Scientific, MA, United States), diluted in PBS for 10 min, and washed with PBS for 5 min. For immunostaining, antigen retrieval was performed with sodium citrate buffer (10 mM sodium citrate, 0.05% Tween 20, and pH 6.0), prepared fresh on the day of use. Sections were incubated in pre-warmed sodium citrate buffer at 65°C for 10 min using a water bath (Fisher Scientific, Isotemp^®^, GPD 10, MA, United States) and plastic Coplin jars. Next, the coplin jars containing the slides were allowed to cool down to RT on the bench for at least 20 min. Sections were then briefly rinsed with ddH_2_O, then washed three times with PBS for 5 min each, blocked in 5% BSA in PBS at RT for 1 h, and then incubated in AffiniPure Fab Fragment goat anti-mouse IgG (H + L) (#115-007-003, 1:100, Jackson ImmunoResearch, PA, USA) diluted in PBS at RT for 30 min. Sections were then incubated overnight at 4°C in a humidified chamber with anti-PAX7 mouse antibody (PAX7-b, AB 528428, 1:100, DSHB, IA, USA) and anti-PCNA rabbit antibody (CPTC-PCNA-3, AB 2888965, 1:25, DSHB, IA, USA) and diluted in 5% BSA in PBS. The following day, the sections were washed with 0.1% Triton-X-100 in PBS for 5 min and washed once with PBS for 5 min before incubation with Alexa Fluor™ 488 goat anti-mouse IgG1 (#A-21121, #A-10667, 1:400, Invitrogen, MA, United States) and Alexa Fluor™ 555 donkey anti-rabbit IgG (H + L) (#A-31572, 1:400, Invitrogen, MA, United States) or Alexa Fluor™ 555 donkey anti-mouse IgG (H + L) (#A-21426 1:400, Invitrogen, MA, United States) diluted in 5% BSA or 0.1% blocking buffer in PBS at RT for 1 h. Sections were then washed with 0.1% Triton-X-100 in PBS for 5 min before being stained with DAPI (4′,6-diamidino-2-phenylindole dihydrochloride) (#D1306, 2 μM, Invitrogen, MA, United States) or NucBlue Live Cell Stain Ready Probe (Hoechst 33342, Invitrogen, MA, United States) diluted in PBS for 10 min, then washed two more times in PBS for 5 min, before incubating with TrueBlack^®^ Plus to quench autofluorescence (#23014, 1:40, Biotium, CA, United States) for 1 h, and washed again in PBS for 5 min. Finally, sections were mounted with VECTASHIELD^®^ PLUS Antifade Mounting Medium (H-1900, Vector Laboratories, CA, United States) or fluorescent mounting medium (#S3023, DAKO, Denmark) and imaged on a fluorescence digital microscope (EVOS M5000, Invitrogen, MA, United States) or fluorescence microscope (ZEISS Axio Observer Z1 microscope, Jena, Germany). Quantification of Pax7+ and PCNA + cells was conducted manually. Quantification of myofiber morphological parameters (size and central nucleation) was conducted using software SMASH (Smith and Barton, 2014).

Proliferating MuSCs were seeded at 18,000–20,000 cells/well in 12-well plates and grown on coverslips (12 mm) for 3 days. MuSC for immunostaining of differentiation experiment (n = 5 in each group) were grown on 96-well plates with seeding 10,000 cells/well, and differentiation was induced after 24 h by medium without serum and allowed to differentiate for another 2 days. MuSCs were fixed with 4% PFA for 10 min and washed 3x with PBS-tween (PBS-T), followed by permeabilization with 0.1% Triton-X-100 for 10 min. Samples were blocked in 1x blocking buffer (#ab126587, Abcam, United Kingdom) in PBS-T for 30 min. Proliferation cells were stained with primary antibodies PAX7 (1:10, PAX7-b, AB 528428, 1:100, DSHB, IA, USA), SDC4 (1:1000, GenScript), or, in the case of differentiated MuSCs, desmin (#ab8592, 1:80, Abcam, United Kingdom) prepared in 0.1% blocking buffer (#ab126587, Abcam, United Kingdom) and incubated overnight at 4°C. The next day, cells were washed twice with PBS-T and incubated with goat anti-mouse IgG, IgM, and IgA (H + L) secondary antibodies conjugated with Alexa Fluor™ 488 (1:400, #A-10667, Invitrogen, MA, United States), Alexa Fluor™ 555 donkey anti-mouse IgG (H + L) (#A-21426, 1:400, Invitrogen, MA, United States), and goat anti-rabbit IgG (H + L) cross-adsorbed secondary antibody conjugated with Alexa Fluor™ 546 (1:400, #A-10667, Invitrogen, MA, USA) and NucBlue Live Cell stain ready probe (Hoechst 33342, Invitrogen, MA, United States) for 2 h at RT. The samples were washed with PBS-T before being transferred onto a microscope slide and mounted using the fluorescent mounting medium (#S3023, DAKO, Denmark) in the case of coverslip glass samples. The slides or wells were examined by fluorescence microscopy analysis (ZEISS Axio Observer Z1 microscope, Jena, Germany), and images were contrast-adjusted using ImageJ (NIH, MD, United States).

### 2.8 Statistical analysis and software

For quantification of Western blots, ImageQuant TL 10.2–499 (Cytiva, GE Healthcare Life Sciences, MA, US) was used, with the background method of rolling ball (radius 2). All quantifications of the bands generated from the Western blots were displayed as the mean ± SEM (standard error of mean, n = 4–5). The statistical analyses of RT-qPCR (n = 7–8) and Western blots (n = 4–5) were performed in Graph Pad Prism version 10.4.0 (GraphPad Software, La Jolla, CA, USA), using nested *t*-test with Welch correction for RT-qPCR and *t*-test with Welch correction for Western blots. Statistical significance was considered *p*-values <0.05, as indicated in each figure. Explorative multivariate analysis by principal component analysis (PCA) was performed on proteomics data, both with and without normalization, using MATLAB 2022b (The MathWorks, Inc, Natick, MA, USA). In addition, explorative univariate analysis was conducted using Welch’s *t*-test for all detected proteins.

## 3 Results

### 3.1 Characterization of metabolic changes and myogenesis in WB *in vivo*

#### 3.1.1 WB myopathic birds show metabolic changes

Previous studies have linked the importance of energy metabolism to the onset of WB development. We, therefore, investigated the salt-soluble proteins (proteins involved in metabolism and cellular processes) of the *pectoralis major* muscle from affected (N = 12) and non-affected (N = 12) animals by label-free quantitative MS-based proteomic analysis.

**FIGURE 1:**
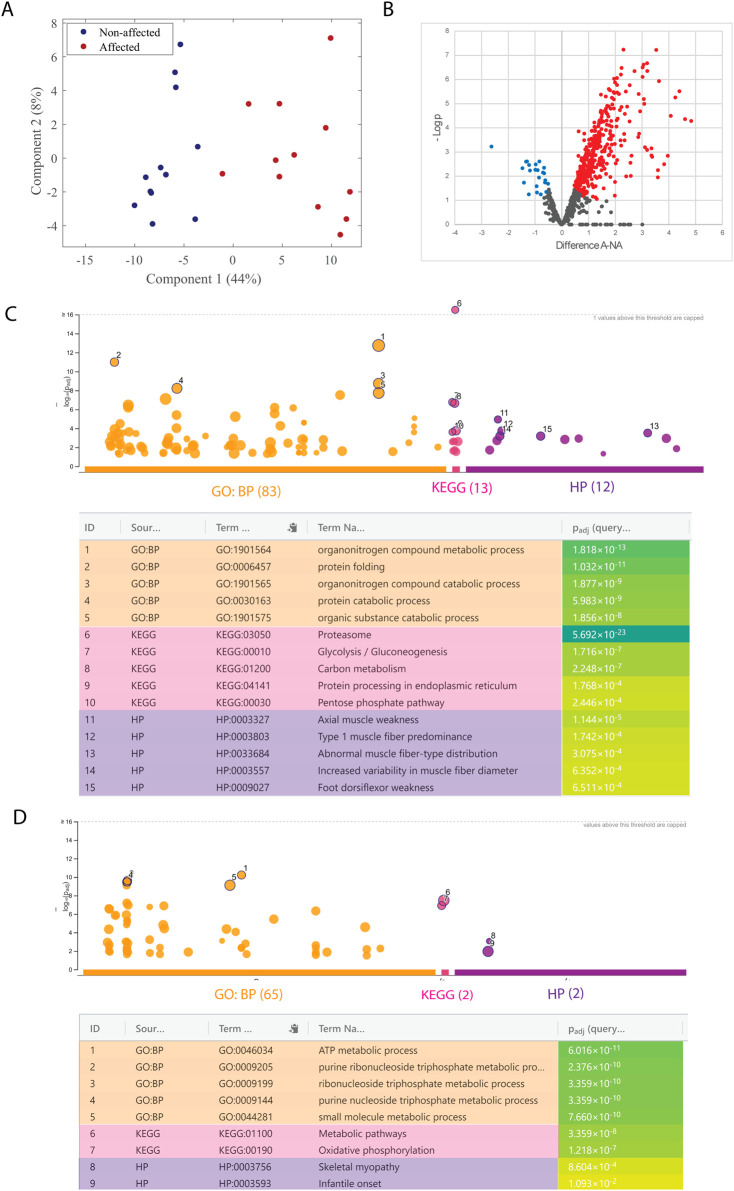
Mass spectrometry-based proteomic analysis of salt-soluble proteins. **(A)** Score plot from the PCA analysis of affected (red; A) and non-affected (blue; NA) samples. **(B)** Volcano plot of fold change (LFQ intensity) in 12 biological replicates for significantly regulated proteins. The plot is represented as a function of statistical significance (Welch’s *t*-test) between affected (A) and non-affected (NA) samples. *X*- and *Y*-axes show protein ratio (log2 change) in A and NA samples and *p*-value (−log10), respectively. Each dot represents one protein. Significantly upregulated and downregulated proteins in A samples were colored red and blue, respectively. The false discovery rate and S0 parameters were set to 0.05 and 0.1, respectively. g: GOSt Manhattan plot of upregulated **(C)** and downregulated **(D)** proteins. The *x*-axis shows the GO functional terms colored by data source (Gene Ontology: biological process, GO: BP; Kyoto Encyclopedia of Genes and Genomes, KEGG; Human Phenotype Ontology, HPO). Each colored dot presents a GO term. The *y*-axis is the adjusted −log10 *p*-values. The GO terms with the five lowest *p*-values were highlighted with a circle and listed in the table below the Manhattan plot. *p*-values in the table are color-coded according to significant levels, light green (less insignificant) to blue (highly significant).

In addition, 819 proteins were identified, and PCA analysis showed a clear separation between the two groups along principal component 1 according to the expression of proteins (Figure 1A). Welch’s *t*-test indicated that 414 proteins were differentially expressed between groups, with 23 proteins significantly downregulated and 391 proteins significantly upregulated in the affected samples (Figure 1B). GO enrichment analyses revealed changes in proteins related to metabolic changes. The five enriched GO terms with the lowest *p*-value for upregulated and downregulated proteins are highlighted in the Manhattan plots and listed in the table (Figures 1C, D, respectively). In upregulated terms such as glycolysis, abnormal muscle fiber-type distribution, and increased variability in muscle fiber diameter. On the other hand, downregulated terms highlighted oxidative phosphorylation, skeletal myopathy, or metabolic pathways. All significantly enriched GO terms from upregulated and downregulated proteins are shown in Supplementary Tables S1, S2, respectively.

#### 3.1.2 Muscle fibers and myogenic markers are altered in WB myopathy

**FIGURE 2:**
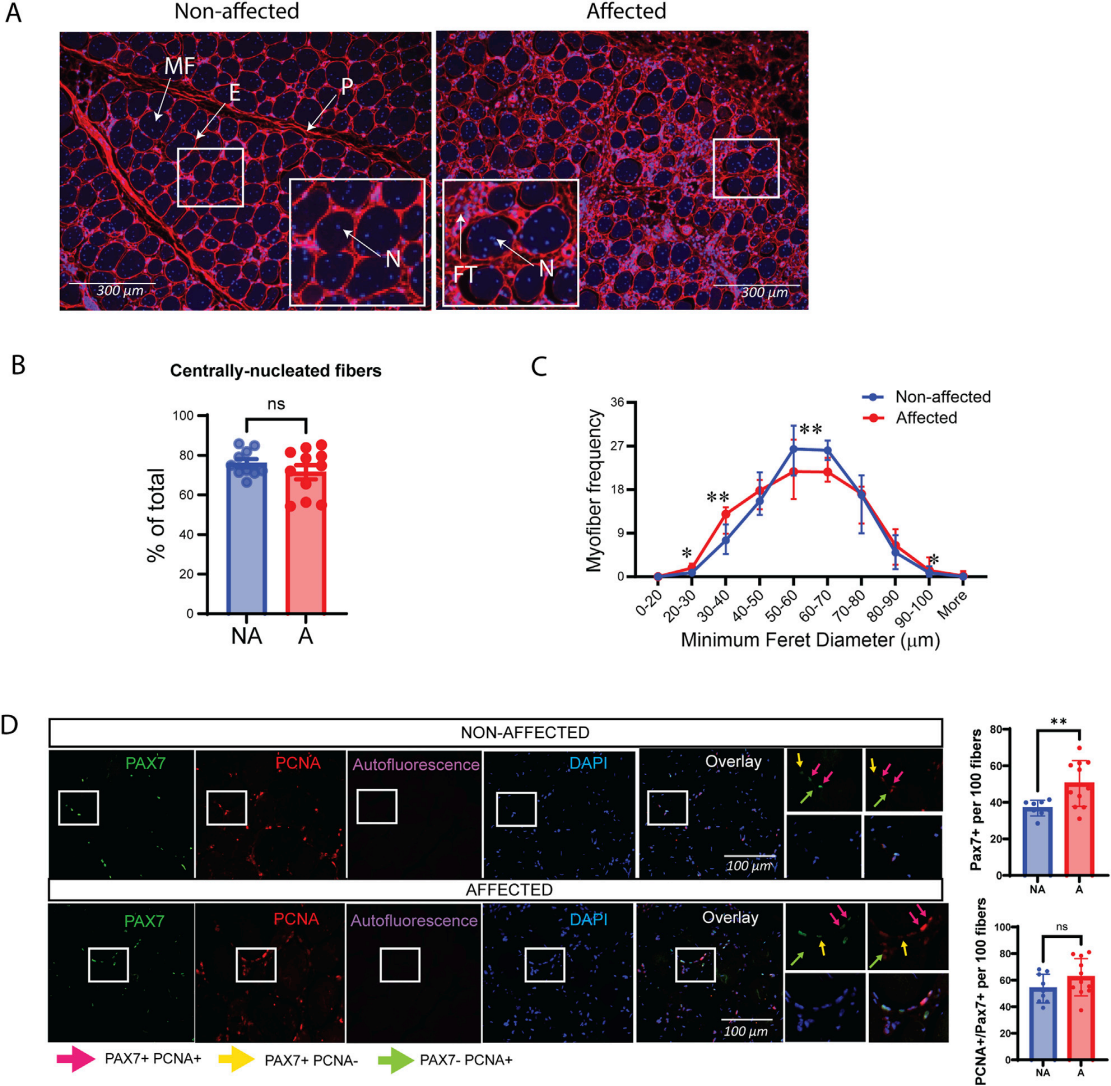
Altered muscle fiber sizes in affected chicken muscle sections and PAX7. **(A)** Representative images of DAPI (blue)/WGA (red) counterstaining of nuclei and muscle fibers in chicken muscle sections. A marked increase in WGA staining (fibrosis) is observed in affected muscle sections. Smaller fibers can also be observed in affected muscle sections by visual inspection. Magnification: ×10 with a fluorescent microscope. P, perimysium; E, endomysium; MF, muscle fiber; FT, fibrotic tissue; N, nuclei. **(B)** Bars represent quantification of centrally nucleated myofibers of affected and non-affected samples images presented in **(A)**. **(C)** Quantification of images in **(A)** show minimum Feret diameter distribution in affected and non-affected samples. **(D)** Representative images of PAX7/PCNA co-immunostaining in chicken muscle sections (left). Quantification of images shows that total PAX7 expressing satellite cells per 100 fibers are significantly increased in animals affected by WB; however, the fraction of proliferating PAX7+ cells are unchanged. A total of 10 random fields were quantified per image/animal. Pink arrows indicate PAX7+PCNA+, proliferating myoblasts. Yellow arrows indicate PAX7+PCNA-, non-proliferating myoblasts, and green arrows indicate proliferating cells that are not myoblasts (Pax7-PCNA+). Scale used was 100 µm. Significance was marked as ns > 0.05; *p ≤ 0.05; **p ≤ 0.01 with N = 10 affected and 11 non-affected. A; affected, NA; non-affected.

Further characterization of the skeletal muscle using WGA staining confirmed extensive fibrosis in WB-affected chickens and widespread central nucleation of myofibers (Figure 2A). Interestingly, the vast majority of fibers were centrally nucleated (~80%), which did not differ between groups (Figures 2A, B), indicative of active degeneration/regeneration regardless of the extent of WB pathology. In affected individuals, the muscle fibers were generally smaller, as shown by a shift toward smaller minimum Feret diameters (20–40 µm). However, there was also an increase in very large fibers (90–100 µm) compared to non-affected individuals (Figure 2C).

Skeletal muscle regeneration relies on a population of locally resident MuSCs, which express the paired box transcription factor family member Paired box 7 (PAX7) (Olguín and Pisconti, 2012; Yin et al., 2013). Co-immunostaining with the proliferation marker proliferating cell nuclear antigen (PCNA) showed that, although the number of PAX7-positive satellite cells was higher in affected samples, the ratio of those that were actively proliferating (PAX7+PCNA+) was the same between affected and non-affected samples, suggesting a mild defect in MuSC proliferation *in vivo* (Figure 2D).

Consistently, RNA sequencing gene expression analysis of muscle tissue samples showed significant differences in several factors important for myogenesis in affected vs. non-affected chicken breast samples. MuSC markers Paired box3 (*PAX3*), *PAX7*, and myogenic factor 5 (*MYF5*), as well as the early myogenic differentiation marker myogenin (*MYOG*), were all upregulated in affected muscles compared with non-affected ones (Supplementary Figure S1A). Although not significant, we observed the same trend for PAX7 and MYOG using RT-qPCR (Supplementary Figures S1B–D).

### 3.2 Exploration of SC proliferation and differentiation

#### 3.2.1 MuSC proliferation is impaired

**FIGURE 3:**
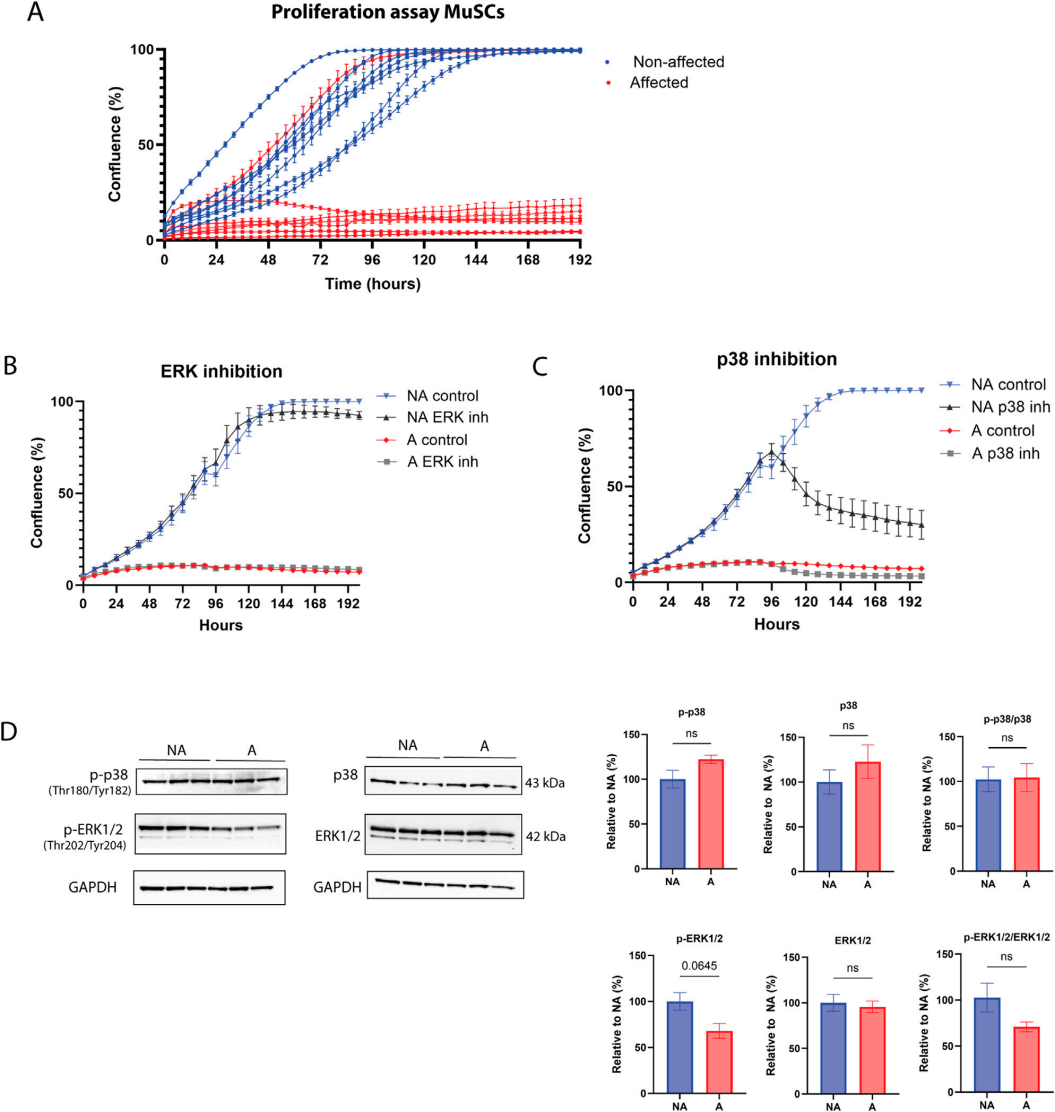
Proliferation disruption and MAPK signaling of WB MuSCs. **(A)** Incucyte S3 live cell proliferation assay (10x) of primary MuSCs isolated from affected and non-affected animals. Compared to affected cell lines (except one outlier SC cell line), all non-affected cell lines reached 100% confluence. **(B)** MuSCs treated with MEK/ERK inhibitor and **(C)** p38 inhibitor, **(D)** protein p38, ERK1/2, their phosphorylated forms, phospho (Thr180/Tyr182)-p38 and phospho (Thr202/Tyr204)-ERK1/2, and their ratios show no difference between WB affected (A) and non-affected (NA) group. Raw data for each condition (N = 3) were normalized to reference protein GAPDH and present it as a relative value to the non-affected group. Significance level was calculated by unpaired *t*-test with Welsh correction (ns, *p* > 0.5).

The data described so far suggests that a defect in myogenesis may be associated with tissue degeneration in the *pectoralis major* of chickens affected by WB. To further investigate a potential cell-autonomous role for MuSCs in the pathogenesis of WB, we isolated primary MuSCs from the *pectoralis major* of affected and non-affected individuals and expanded them in culture using established isolation and culture conditions (Yoshioka et al., 2020). Upon observing cell proliferation (Figure 3A), it became evident that the cells sourced from WB-affected muscles demonstrated impaired proliferation. Most of these cells failed to achieve more than 50% confluency before the cessation of cell growth. Two key signaling pathways regulating cell proliferation are the ERK and p38 pathways (Zhang and Liu, 2002). When these signaling pathways were inhibited (Figures 3B, C), the response was similar in both non-affected and affected chickens, suggesting these pathways were not impaired. Interestingly, inhibiting the ERK pathway did not impact cell proliferation in MuSCs (Figure 3B), while inhibiting p38 dramatically stopped proliferation in non-affected MuSCs (Figure 3C). Protein expression of p38 and ERK1/2 and their phosphorylated forms showed no statistical difference between groups; however, phosphorylated ERK1/2 showed a tendency to be downregulated in affected chickens (Figure 3D). These observations suggest that although the p38 MAPK signaling pathway is important for cell proliferation in chickens, this signaling pathway does not appear to be fully impaired in WB-affected cells.

**FIGURE 4:**
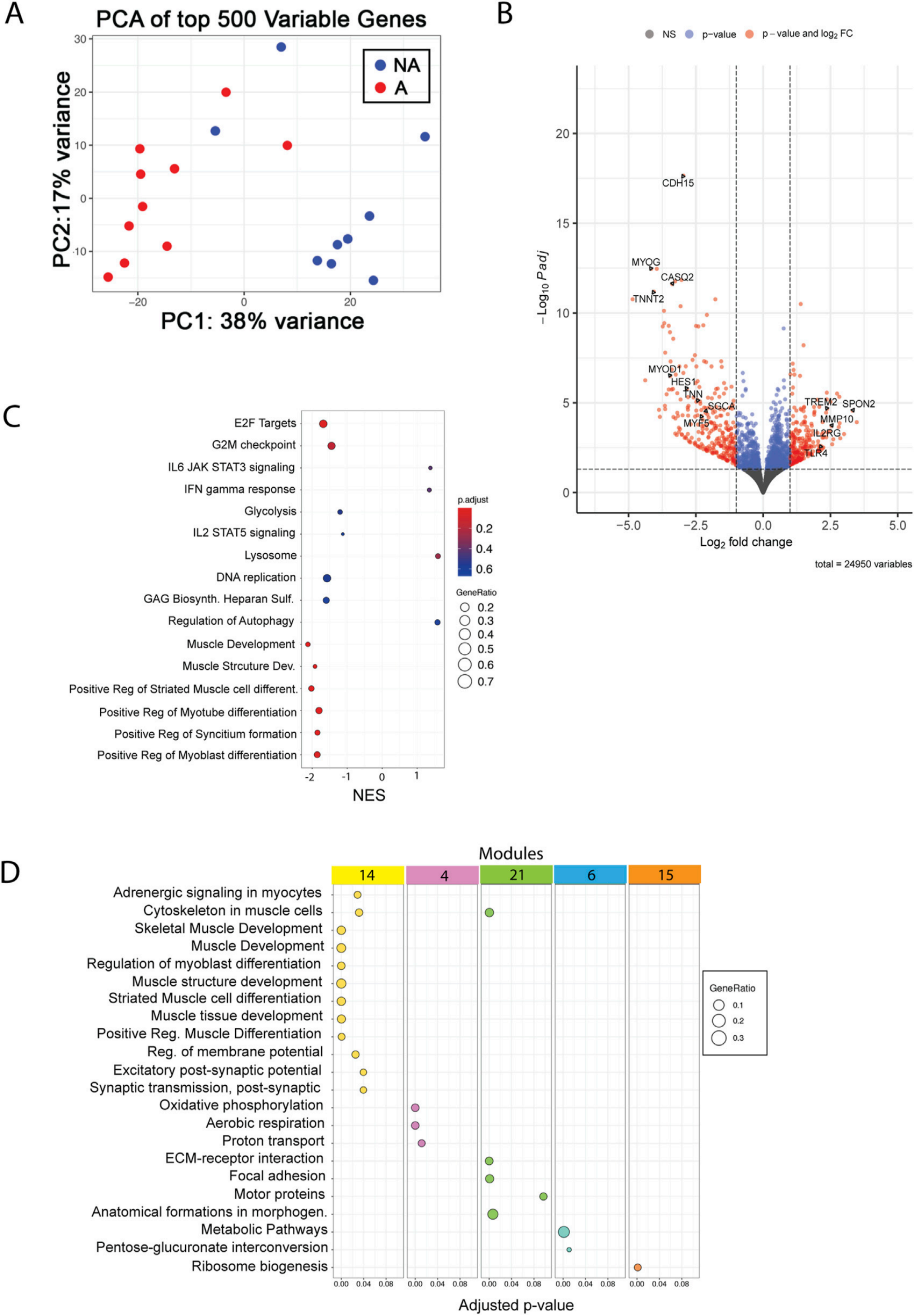
RNA-seq analysis of MuSCs. **(A)** PCA plot of top 500 variables genes between affected and non-affected chickens. A, affected; NA, non-affected **(B)** Volcano plot with selected genes showing the adjusted p-values and the log2 fold change (FC) values of genes. DEGs, indicated by the red dots, were identified as genes with an adjusted p-value of ≤ 0.05 and |log2 FC| between −1 and 1, and blue dots indicate only p-value. X-axis shows downregulation in the negative range of axis, and genes in the positive range are upregulated. **(C)** GSEA shows the top 16 pathways sorted by the normalized enrichment score, adjusted p-value (p-adjust), and gene ratio. **(D)** Functional enrichment analysis of the top five modules identified by network analysis.

To gather further insights into the underlying molecular causes of the striking differences observed in MuSC proliferation *in vitro* between affected and non-affected animals, we carried out transcriptomic analysis using RNA extracted from proliferating MuSCs. Surprisingly, the number of differentially expressed genes was relatively small, with 232 upregulated and 278 downregulated genes (Figure 4A) in the affected *versus* non-affected samples. Consistently, PCA analysis showed partial overlap between groups (Figure 4B); however, GSEA identified several terms that were differentially expressed between affected and non-affected MuSCs. The most highly significant function, with a negative normalized enrichment score (NES), downregulated in affected *versus* non-affected MuSCs samples, was related to proliferation (E2F targets and G2/M checkpoint, Figure 4C), as well as terms related to myogenesis (muscle structure development, cell, myotube, and myotube differentiation) (Figure 4C). In contrast, functions related to metabolisms appeared both upregulated (e.g., regulation of autophagy and lysosome) and downregulated (glycolysis), albeit overall pointing toward an anabolic trend. Finally, among the most significant trends were also those related to a pro-inflammatory response, which were mostly upregulated (e.g., interferon-gamma response and IL-6 signaling) (Figure 4C).

Investigation of senescence signs and markers of WB-affected MuSCs showed that apart from CDKN1a (p21), neither the genes of the senescence-associated secretory program (Supplementary Figure S2A) nor other cell cycle arrest markers (Supplementary Figures S2C–E) were differentially expressed in cells from affected chickens compared with cells from non-affected chickens. On the other hand, downregulation of *TP53 (* Supplementary Figures S2B, C *)*, *LMNB2* (Supplementary Figure S2B **)**, and *Ki67* (Supplementary Figure S2F) was observed in affected proliferating MuSCs. Inspection of nuclei morphology revealed a decreased perimeter of nuclei and increased area and circularity of nuclei in the affected cells (Supplementary Figure S2G).

Finally, network analysis identified five gene modules that were differentially enriched in affected vs. non-affected MuSCs. These modules again mapped to functions related to muscle development (Figure 4D, module 14), metabolisms (Figure 4D, modules 4, 6, and 15), and cell adhesion (Figure 4D, module 21), further supporting the findings previously described in the pathogenesis of WB.

#### 3.2.2 Myogenesis in WB MuSCs is reduced, but differentiation remains intact

**FIGURE 5:**
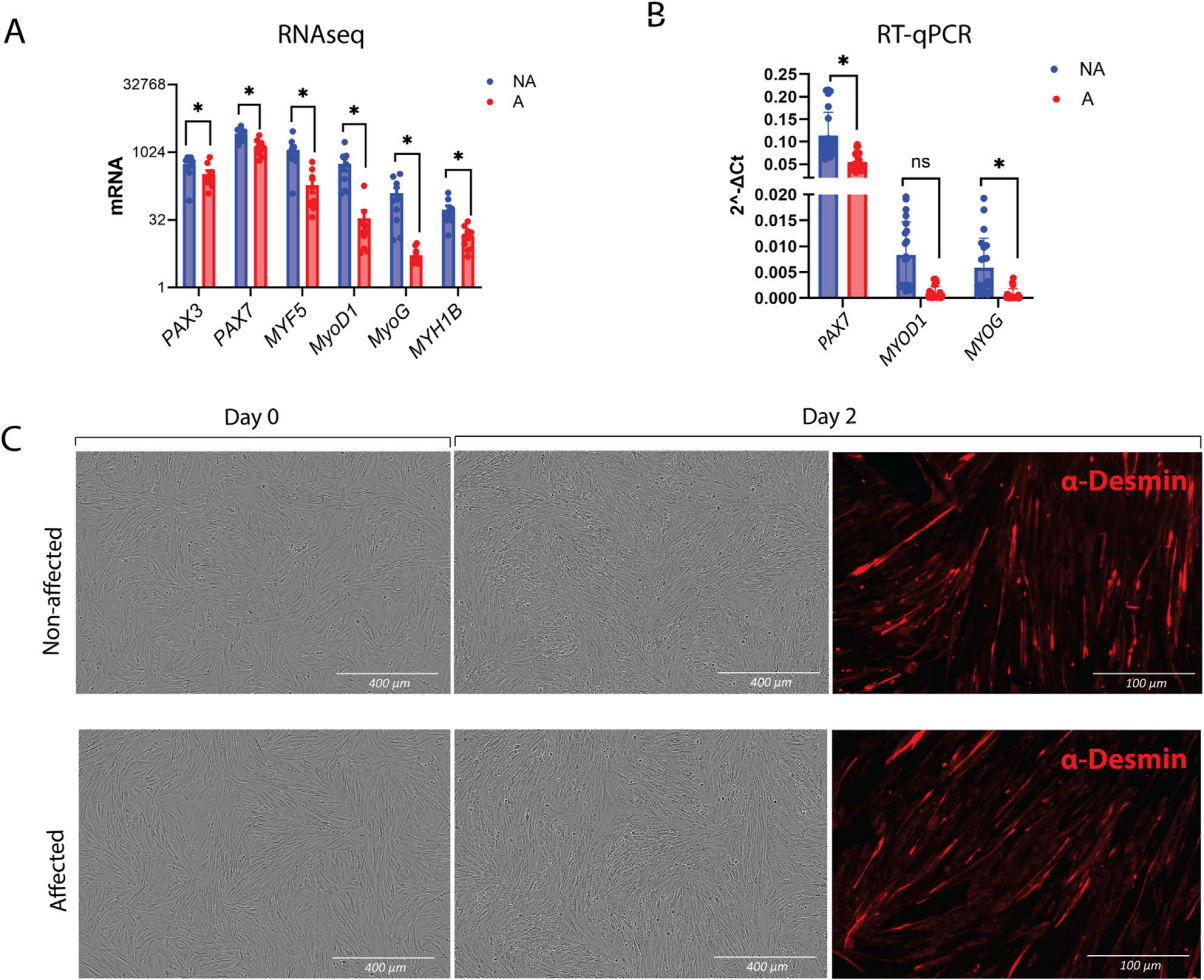
Myogenesis and differentiation in MuSCs. **(A)** Gene expression of myogenesis markers obtained by RNA-seq and **(B)** verification of *PAX7*, *MYOD1*, and *MYOG* by RT-qPCR. The data are presented as the fold change average relative to the mean of non-affected WB ± SEM. Comparisons between the groups were analyzed using the *t*-test with Brown–Forsythe and Welsh correction; *p ≤ 0.05 **(C)** Differentiation assay showing intact differentiation in affected MuSCs after 2 days. Chicken NA and A MuSCs were seeded in roughly 80% confluency. Differentiated MuSCs were immunostained after 2 days with Desmin and secondary antibody Alexa Fluor™ 555 donkey anti-mouse IgG (Right panel). A, affected; NA, non-affected.

Since the downregulation of myogenesis was the most striking transcriptomic signature displayed by MuSCs isolated from affected chickens compared with those from non-affected chickens, we carried out RT-qPCR-based validation of genes that play a key role in myogenesis. As already observed in RNAseq (Figure 5A), *PAX3* and *PAX7* showed a modest, although statistically significant, decrease in affected samples, while *MYF5*, *MYOD*, and *MYOG* showed a trend toward a greater decrease, which was validated by RT-qPCR *MYOG* and *PAX7* by RT-qPCR (Figure 5B).

Since the gene expression data suggested a differentiation defect, we sought to test whether cells from WB-affected chickens were inherently differentiation-defective. The differentiation assay was performed by seeding the cells at high density and immediately inducing them to differentiate without prior expansion. Surprisingly, MuSCs from both affected and non-affected chickens were able to form myotubes, as visualized by desmin staining (Figure 5C).

#### 3.2.3 Syndecan-4 decreases the proliferation rate, and its shedding increased during WB

**FIGURE 6:**
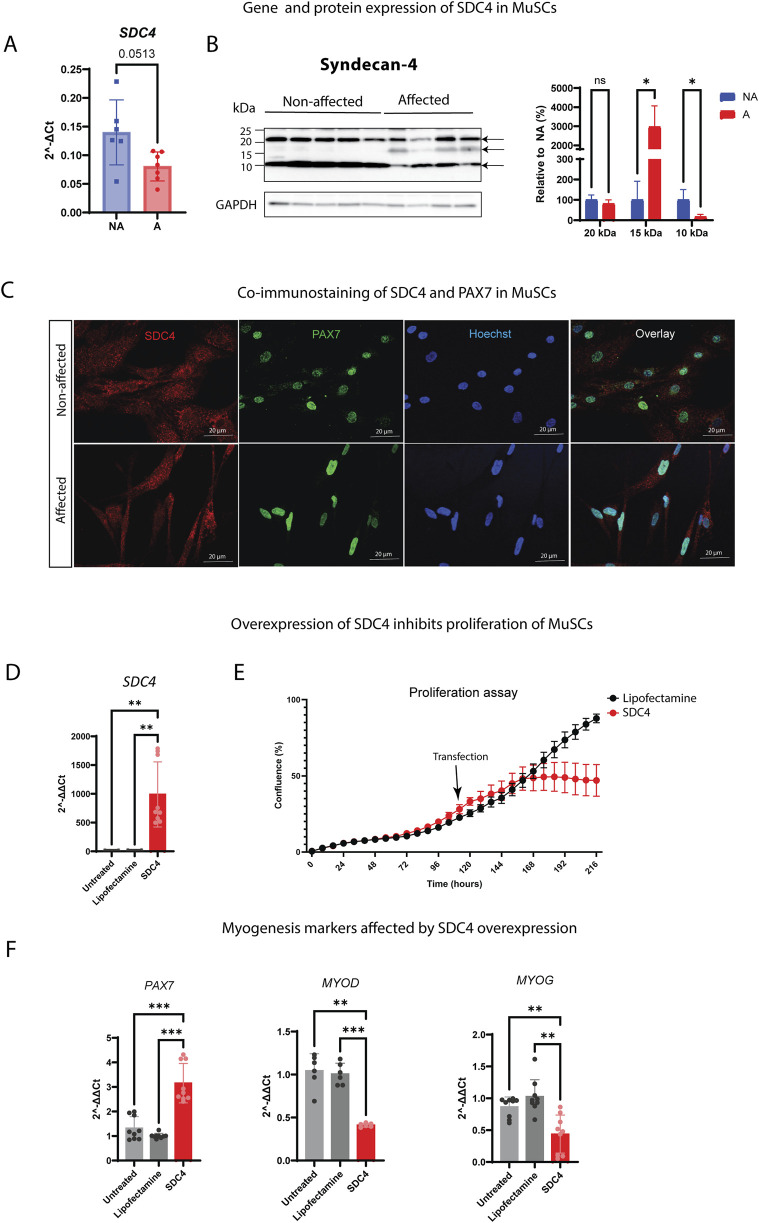
Expression and overexpression of SDC4 in chicken MuSCs. **(A)** Bars show the relative gene expression of SDC4 in non-affected (NA) and affected (A) chicken MuSCs (n = 8 chickens in each group) assessed by RT-qPCR. The data are presented as an average of eight chickens in triplicates. The two groups were compared using an unpaired *t*-test with Welch’s correction. **(B)** Levels of the syndecan-4 core protein (22 kDa) and two remaining syndecan-4 fragments after shedding (10 and 15 kDa) in non-affected (n = 5) and affected (n = 4) MuSCs were quantitated using ImageQuant TL and normalized to the loading control (GAPDH) and the average of NA group. Data were analyzed using an unpaired *t*-test with Welch’s correction and presented as a relative percentage to NA. Arrows indicate the three specific syndecan-4 bands (10, 15, and 20 kDa) confirmed by blocking experiments of the SDC4 antibody (data not shown) (ns *p* > 0.05; *p ≤ 0.05). **(C)** Co-immunostaining of SDC4 (red) and PAX7 (green) in proliferating affected and non-affected chicken MuSCs. Additionally, the panel shows staining of nuclei (blue) and overlay of the channels. **(D)** Gene expression levels of SDC4 in SDC4-transfected MuSCs vs. lipofectamine-treated and non-transfected/untreated MuSCs (controls). **(E)** Proliferation assay showing decreased proliferation of SDC4 overexpressing MuSCs. The black arrow indicates the time point of transfection (4 days of proliferation). Lipofectamine was used as a control. N = 3 individual WB non-affected chickens. **(F)** Gene expression levels of the myogenic markers *PAX7*, *MYOD1*, and *MYOG* in SDC4 overexpressing MuSCs. Untreated and lipofectamine-treated MuSCs were used as controls. Three individual animals measured in technical triplicates. Significant differences were detected using one-way ANOVA with Brown–Forsythe and Welsh correction (**p ≤ 0.01; ***p ≤ 0.001).

We have previously shown that SDC4 is a crucial regulator of MuSC homeostasis and function (Pisconti et al., 2012; Rønning et al., 2015; Velleman and Song, 2017; Keller-Pinter et al., 2018). When analyzing the relative gene expression of SDC4 in isolated MuSCs, we observed a slight, although not statistically significant, downregulation in the affected cells compared to the non-affected ones (Figure 6A). We have previously developed specific antibodies targeting the cytoplasmic part of the chicken SDCs (Pejšková et al., 2023). Interestingly, the band representing the core protein (20 kDa) and the remaining 10–17 kDa fragments, left after shedding, differed greatly between the groups. We observed that the 17 kDa fragment increased in the affected cells, while the 10 kDa band decreased, indicating a different shedding pattern (Figure 6B, full-length immunoblot is shown in Supplementary Figure S3A). We could not detect a difference in the intracellular localization pattern of SDC4 when we co-stained cells with PAX7 (Figure 6C).

To further study the mechanism of SDC4 *in vitro*, we overexpressed SDC4 in non-affected chicken MuSCs (Figure 6D), which confirmed the involvement of SDC4 in cell proliferation since we observed a reduced cell growth of transfected cells compared to non-transfected controls (Figure 5E). However, this reduction was not linked to p38 MAPK or its phosphorylation (Supplementary Figure S4). Likewise, the overexpression of SDC4 influenced gene expression levels of *PAX7*, *MYOD*, and *MYOG* (Figure 6F).

Finally, we analyzed the gene expression levels of the other SDCs and observed that the gene expression of SDC1–3 was upregulated although not significantly for SDC1 and SDC3 (Supplementary Figure S3B). When we examined their protein levels, the SDC1–3 band pattern differed greatly between the groups, showing increased shedding in affected animals compared to non-affected ones (Supplementary Figure S3B), similar to what we observed for SDC4 (Figure 6B). Additionally, SDC4 overexpression in non-affected MuSCs led to increased gene expression of SDC1 and SDC2, whereas SDC3 remained unchanged, suggesting that SDC4 is able to modulate levels of other SDC family members in MuSCs (Supplementary Figure S3C).

### 3.3 Blocking peptides derived from SDC4 ectodomain reduced shedding

**FIGURE 7:**
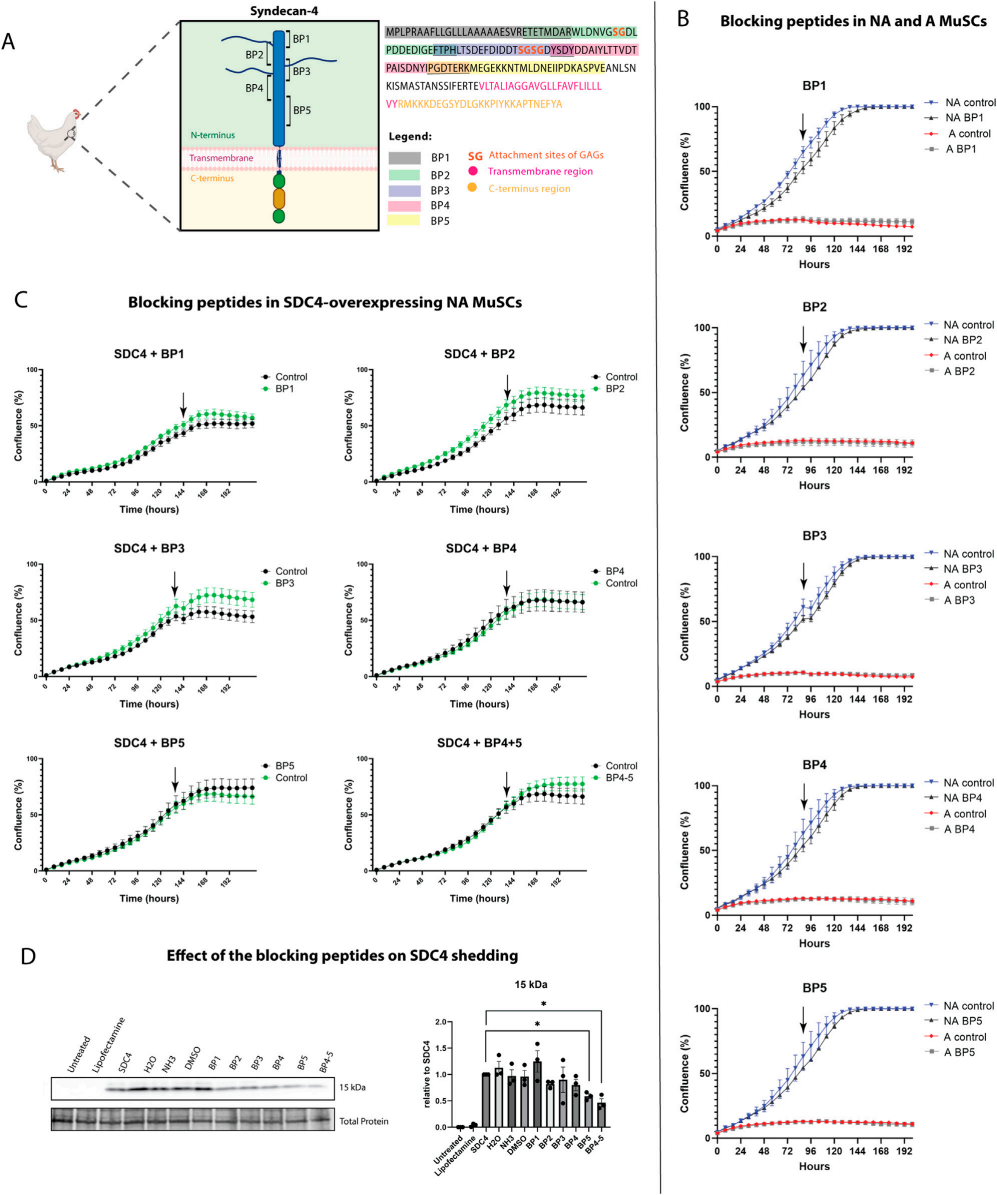
Effect of syndecan-4 derived blocking peptides on MuSC proliferation and shedding. **(A)** Schematic illustration of chicken SDC4 with its extracellular domain showing overlapping blocking peptides. Full sequence of chicken SDC4 showing exact amino acids of each blocking peptide and their overlap (underlined). The sequence also features SDC4 transmembrane domain (pink), C-terminus (light orange), and GAG attachment sites (dark orange). Illustration used partially image source from Biorender.com. **(B)** Effect of syndecan-4-derived blocking peptides on the proliferation of affected and non-affected MuSCs. **(C)** Effect of syndecan-4 derived blocking peptides on the proliferation of SDC4-overexpressing MuSCs. Black arrows indicate the time point of treatment with the respective blocking peptides. Lipofectamine was used as a control at the same final concentration. **(D)** Immunoblot of the 15 kDa shed SDC4 fragment in MuSCs treated with or without the respective blocking peptides. The levels were quantified using ImageQuant TL, and values were normalized to total protein LI-COR staining (loading control) and SDC4-overexpressing MuSCs without any peptide treatment (set to 1). For comparison, untreated MuSCs and the lipofectamine control are presented. Significant differences were detected using an unpaired *t*-test with Welch’s correction. N = 3 for individual non-affected chickens (*p ≤ 0.05).

To investigate whether SDC4 shedding is involved in the regulation of MuSC proliferation *in vitro*, we developed five overlapping blocking peptides representing the extracellular part of chicken SDC4 (Figure 7A). When incubated with the MuSCs, the SDC4-derived blocking peptides (BP1-5) did not show any significant effect on proliferation, neither on non-affected nor affected cells (Figure 7B). However, when the peptides were incubated with non-affected MuSCs overexpressing SDC4, an increase in the growth rate was observed, particularly in the presence of BP3 or the combination of BP4+5 (Figure 7C). Furthermore, immunoblot analysis showed that at least BP5 appeared to reduce SDC4 shedding (Figure 7D). Notably, SDC4 overexpression could not be achieved in affected MuSCs due to their low proliferation rate.

## 4 Discussion

### 4.1 Skeletal muscle regeneration in WB chickens *in vivo*

This study aimed to investigate the underlying causes of the pathological changes in skeletal muscle in chickens with WB, specifically focusing on the role of SDC function. We have previously identified altered SDC expression and function associated with the extensive ECM remodeling in chicken WB myopathy (Pejšková et al., 2023). The chicken breed Ross 308 investigated in this study developed WB myopathy at relatively high rates and showed signs of degenerative pathologies, such as central nucleation, regardless of the disease severity. Interestingly, even the group classified as ‘non-affected’ in our study, despite lacking extensive fibrosis, includes birds whose breast muscle shows centrally nucleated myofibers and, occasionally, necrotic myofibers and regions of inflammation. There were no differences in the number of centrally nucleated fibers between affected and non-affected WB birds. Although the percentage of centrally nucleated fibers in our study was similar between groups, other studies have shown that these fibers, defined by nuclei positioned at the center of the cytoplasm, are markers of muscle regeneration (Gutpell et al., 2015). They are commonly observed in patients with muscular dystrophies and their animal models (Folker and Baylies, 2013; Sihvo et al., 2014; Roman and Gomes, 2018), suggesting a potential genetic predisposition to myopathy in all Ross 308 chickens.

A hallmark of several muscular disorders, including wooden breast myopathy, is myofiber size heterogeneity, often resulting from continuous cycles of degeneration/regeneration (Soglia et al., 2016). Our histological analysis revealed a significant increase in small and large muscle fibers in affected chickens compared to medium muscle fiber sizes of non-affected chickens. This observation is partially consistent with previous research, showing an increase in small myofibers in WB (Meloche et al., 2018). Von Maltzahn et al. (2013) demonstrated that muscles of *Pax7* ^*−/−*^ mice showed myofibers with approximately 50% fewer nuclei and significantly smaller fiber diameters. However, in our case, we observed increased *PAX7* gene expression, as well as a higher number of total PAX7 expressing satellite cells per 100 fibers. Additionally, *PAX3*, *MYF5*, *MYOG*, and *MYH1B* were upregulated, indicating an expansion of the MuSC pool and maintained differentiation potential. This contrasts with previous findings by Daughtry et al. (2017), who showed that greater muscle hypertrophy correlates with a decrease in MuSCs and their impaired function.

Fibrosis and metabolic alterations are deeply interconnected with the progression and severity of muscle disorders (Lake and Abasht, 2020; Lake et al., 2022; Chen et al., 2023). Our proteomic data highlighted metabolic changes in affected samples linked to muscle abnormalities and significant disruptions in glycolysis, lipid, and amino acid metabolism. Mitochondrial processes play a crucial role in maintaining cellular homeostasis and skeletal muscle health, and their dysfunction is a key feature of muscle disorders such as muscular dystrophy, sarcopenia, and cachexia—all marked by muscle mass loss, reduced fiber size, decreased strength, and fibrosis (Hasegawa et al., 2021). We observed downregulation of proteins related to respiration and mitochondrial function, and GO terms of interest included proteins related to muscle fiber type, distribution, diameter variability, oxidative phosphorylation, and skeletal myopathies, suggesting mitochondrial dysfunction and oxidative stress involved in our affected chickens (Supplementary Table S2) and aligning with previous metabolomic analysis in WB chickens (Wang et al., 2023). Similar findings have been highlighted in other proteomics studies on white striping myopathy (Kong et al., 2024) and oxidative stress (Carvalho et al., 2023).

### 4.2 Involvement of syndecan-4 in impaired MuSC proliferation

Muscle regeneration involves several stages: necrosis of the injured muscle cells, activation and proliferation of muscle stem cells, differentiation into muscle fibers, remodeling of the muscle tissue, and, finally, maturation of the regenerated fibers. To examine the regenerative capabilities of primary chicken MuSCs, MuSCs were extracted from both affected and non-affected chicken pectoral muscles, and their proliferation was assessed in real time. Primary MuSCs from WB-affected chickens exhibited impaired proliferation but were still able to differentiate and form myotubes, in contrast to previous reports of reduced proliferation accompanied by a loss of differentiation capability (Daughtry et al., 2017; Xu and Velleman, 2023). RNA-seq analysis of the MuSCs identified several functions associated with impaired proliferation (E2F targets and G2/M checkpoint) and DNA replication, supporting our findings of decreased proliferation *in vitro*. Moreover, we also observed the downregulation of development and differentiation markers, such as *PAX7*, *PAX3*, *MYF5*, and *MYOG.* The seemingly conflicting results between our RNA-seq data, which suggest a differentiation defect, and the differentiation assay results, which show that MuSCs from affected animals retain their differentiation potential *in vitro*, can be explained by the fact that delayed proliferation may lead to delayed initiation of the differentiation program due to the community effect (Arnold et al., 2020).

Cellular senescence in skeletal muscle serves multiple functions, and it has been proposed that senescence in MuSCs delays muscle regeneration (Saito and Chikenji, 2021). Furthermore, signs of senescence have been observed in other myopathies in chickens (Wright, 1985; Malila et al., 2020). To investigate whether the impaired proliferation in chicken MuSCs was due to senescence, we examined multiple senescence markers. Senescent cells exhibit characteristic morphological and biochemical changes, including hypertrophy, flattened morphology, incomplete nuclear envelope, and increased activity of biomarkers such as *CDKN2A*, *CDKN1A*, *Ki67*, γH2AX, and SA-β-gal, markers of the senescence-associated secretory phenotype (SASP), such as IL6 and LAMB1, several insulin-like growth factor binding proteins, and MMPs and their inhibitors such asTIMP (Gorgoulis et al., 2019; Hansel et al., 2020; Saito and Chikenji, 2021). Our data showed only partial morphological changes in the proliferating MuSCs, along with an increase in *CDKN1A* mRNA, without any alterations in SASP gene expression. This suggests that the impairment in MuSC proliferation is unlikely to be solely caused by senescence.

Subsequently, we explored whether an impaired response to mitogenic signals might contribute to the reduced proliferation of primary MuSCs from WB-affected chickens. It has been earlier postulated that a possible mechanism for senescence-induced impairment of muscle regeneration is the premature senescence triggered by sustained p38 MAPK activity, leading to stem cell exhaustion (Bernet et al., 2014; Cosgrove et al., 2014; Blau et al., 2015; Saito and Chikenji, 2021), although the involvement of signaling pathways in WB myopathy remains not fully explained. To test this hypothesis, we inhibited the P38 MAPK and MEK/ERK signaling pathways, which are recognized as crucial regulators of cell proliferation, differentiation, and survival (Jones et al., 2001; Jones et al., 2005; Perdiguero et al., 2007). While the inhibition of ERK did not affect the proliferation rate in either of the samples, the inhibition of p38 led to a decrease in proliferation in both affected and non-affected cells, suggesting that neither ERK nor p38 is likely involved in the mechanisms that lead to WB-associated defect in MuSC proliferation. Observation of their protein expression and phosphorylation during WB myopathy showed that p38 remains without changes between affected and non-affected group, and ERK1/2 showed a tendency to decrease phosphorylation in WB-affected MuSCs. However, in our earlier *in vivo* investigation of WB myopathy, we observed the upregulation of the ERK1/2 MAPK signaling pathway (Pejšková et al., 2023), which supports the involvement of the ERK pathway in WB, although not necessarily through its role in myogenesis.

In mammals, all four SDCs are expressed during muscle development (Cornelison et al., 2001; Olguin and Brandan, 2001; Do et al., 2015) and contribute to the regulation of myogenesis and MuSC activity, as supported by multiple studies (Cornelison et al., 2001; Cornelison et al., 2004; Velleman et al., 2004; Pisconti et al., 2012; Rønning et al., 2015; Pisconti et al., 2016). Our RNA-seq from MuSCs isolated from WB-affected chickens revealed the downregulation of several gene terms associated with ECM-receptor interactions, focal adhesion, and GAG heparan biosynthesis, pointing toward the involvement of SDCs. Interestingly, the loss of MAPK signaling in SDC4 knockout mice prevented MuSC activation and proliferation (Jones et al., 2001; Jones et al., 2005; Perdiguero et al., 2007; Karimian et al., 2016), showing SDC4 to be required for satellite cell activation. On the other hand, other studies have shown that silencing or knockdown of *SDC4* resulted in decreased progression of cell cycle (Keller-Pinter et al., 2018) and decreased proliferation (Yan et al., 2014; Velleman et al., 2018; Pham et al., 2023). Interestingly, SDC1 expression is shown to be downregulating SDC4 via ERK1/2 and p38 (Hara et al., 2021). Due to the decreased tendency of the mRNA level of *SDC4* in WB-affected MuSCs and their reduced proliferation rate, we tested whether overexpression of SDC4 would impact proliferation or alter myogenesis. Our results demonstrated a reduction in *MYOD* and *MYOG* gene expression following SDC4 overexpression in NA MuSCs, mirroring the findings observed in the cells isolated from chickens with WB. This suggests that SDC4 overexpression may mimic the impaired myogenesis observed in WB. In contrast, the overexpression of SDC4 led to an increase in *PAX7* mRNA and a reduction in MuSC proliferation, suggesting a role for SDC4 in promoting MuSC self-renewal at the expense of myogenesis. Consistent with our findings, the overexpression of SDC4 in turkey satellite cells has been shown to reduce proliferation after 72 h, and it has been proposed that increased SDC4 expression may lead to the formation of more focal adhesions, thereby reducing cell migration (Song et al., 2011).

### 4.3 Shedding of the SDC4 ectodomain contributes to wooden breast

Although we did not observe any significant changes in the relative gene expression of *SDC4* in proliferating MuSCs, we detected alterations in protein expression, specifically the various SDC4 fragments that remained after shedding. This was also the case for the other SDCs, highlighting the complexity and significance of these molecules in WB myopathy *in vitro*. These findings complement our earlier *in vivo* study, indicating that all SDCs are present during WB and exhibit significant shedding (Pejšková et al., 2023). Although SDC1 has been extensively studied for its shedding and use as a biomarker for several diseases (Bertrand and Bollmann, 2019; Rangarajan et al., 2020), shedding of SDC4 has been previously shown as an important regulator in pathologies such as cardiac dysfunctions (Strand et al., 2013; Strand et al., 2023), diabetes mellitus (Li et al., 2016), osteoarthritis (Bollmann et al., 2021), and cancer (Choi et al., 2010).

The SDC4 ectodomain is critical in interactions with the extracellular matrix, growth factors, and cytokines (Tkachenko et al., 2005). Shedding of this ectodomain affects cell behavior, such as cell migration during tissue repair (Manon-Jensen et al., 2010), although the complete impact of this process is not yet fully understood. We tried to elucidate the function of SDC4 ectodomain shedding by adding custom-made blocking peptides to proliferating non-affected and WB-affected MuSCs, representing specific areas of the SDC4 ectodomain. We could not observe any change in cell proliferation when adding the peptides. However, when adding the BPs to SDC4-transfected MuSCs, we were able to restore some of the proliferation capability, especially using the blocking peptide BP3. BP3 consists of the chicken protein sequence FTPHLTSDEFDIDDT**SGSG**DYSDY. Interestingly, this sequence corresponds to the SDC4 ectodomain, containing two consecutive Ser-Gly (SG) sites reported to be GAG chain regions (Kokenyesi and Bernfield, 1994). Compared to SDC1 and SDC3, SDC4 exhibits only heparan sulfate GAG chains. Heparan sulfates on proteoglycans regulate growth factor signaling by acting as co-receptors, reservoirs, or transporters; therefore, adding BP hindering shedding activity might alter the biological function of syndecan directly or indirectly (Keller-Pinter et al., 2018). It has been shown that several growth factors bind to the SDC4 heparan sulfates, which then act as co-receptors for tyrosine kinase activity (Sarrazin et al., 2011). However, it is important to point out that our BP3 is most likely not able to bind to ligands that are dependent on heparan sulfates/GAGs as BP3 only is a short protein sequence with identical amino acids to a smaller stretch of the SDC4 ectodomain. It is possible that BP3 is competing with other ligands containing similar protein sequences, preventing the cells from undergoing differentiation, which might explain our increase in cell proliferation. Still, we cannot rule out the possibility of peptide uptake into the cell, with GAG chains of other molecules potentially playing a role in this process (Favretto et al., 2014), or effect on other cellular functions, such as signaling pathways, adhesion dynamics, or differentiation. Interestingly, shedding of the core SDC4 protein was significantly reduced by BP5 or a combination of BP4 and BP5, which indicates that at least BP5 contains an MMP-binding (cleavage) site, allowing it to outcompete MMP binding to the SDC4 ectodomain, thereby inhibit shedding of SDC4. This observation corresponds with previously detected cleavage by MMP2 and MMP9 in the human SDC4 ectodomain in lysine 105 (Manon-Jensen et al., 2013) situated in our BP5 region. However, current knowledge about the specific cleavage sites on the chicken SDC4 core protein remains limited. Our findings offer new insights into the cleavage and shedding of SDC4 in chickens. Considering the pivotal role of SDC4 in cellular regulation and disease, additional research is essential to unravel the mechanisms driving myogenesis.

## 5 Conclusion

This study aimed to elucidate the molecular basis of WB myopathy. Our research revealed significant molecular and cellular alterations associated with WB myopathy and indicated a role of SDC4 in its pathogenesis. We showed different shedding patterns of SDC4 in affected and non-affected primary MuSCs and observed reduced growth in non-affected MuSCs during SDC4 overexpression. Using specific blocking peptides, each representing a specific part of the chicken SDC4 ectodomain, we demonstrated increased proliferation and decreased shedding of SDC4 in MuSCs. The increased shedding identified across all members of the syndecan family suggests an involvement of SDCs in the pathogenesis of WB; further investigation into their regulatory mechanisms could provide valuable insights for the development of targeted therapeutic interventions.

## Data Availability

The proteomic data presented in the study are deposited in the UCSD/CCMS - MassIVE Datasets - Mass Spectrometry Repository Dataset List repository, accession number MSV000096414. The RNA seq data presented in the study are deposited in the GEO Accession viewer repository, accession number GSE279699.
