# Supplementary material for: Wooden breast myopathy is characterized by satellite cell dysfunction and syndecan-4 shedding
Source: Front Physiol. 2024 Dec 23;15:1513311. doi: 10.3389/fphys.2024.1513311 (PMC11701147; doi:10.3389/fphys.2024.1513311)
Supplement: Supplementary file 1 [file DataSheet1.pdf]

Supplementary Material

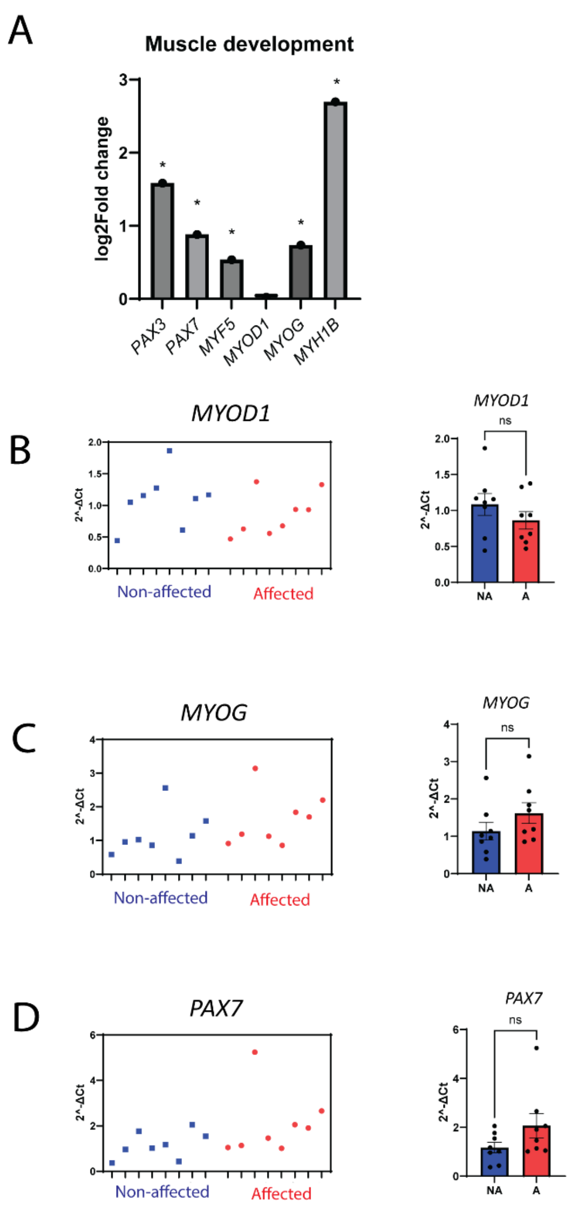

Figure S1.

Gene expression of muscle markers *in vivo*. (A) Gene expression of muscle development markers in whole muscle samples obtained by RNAseq, represented as a log2Fold change in affected samples vs. non-affected. * p-value < 0.05. RT-qPCR plotted in nested and column visualization for **(B)** *MYOD1*, **(C)** *MYOG*, and **(D)** *PAX7*. A, Affected; NA, Non-affected.

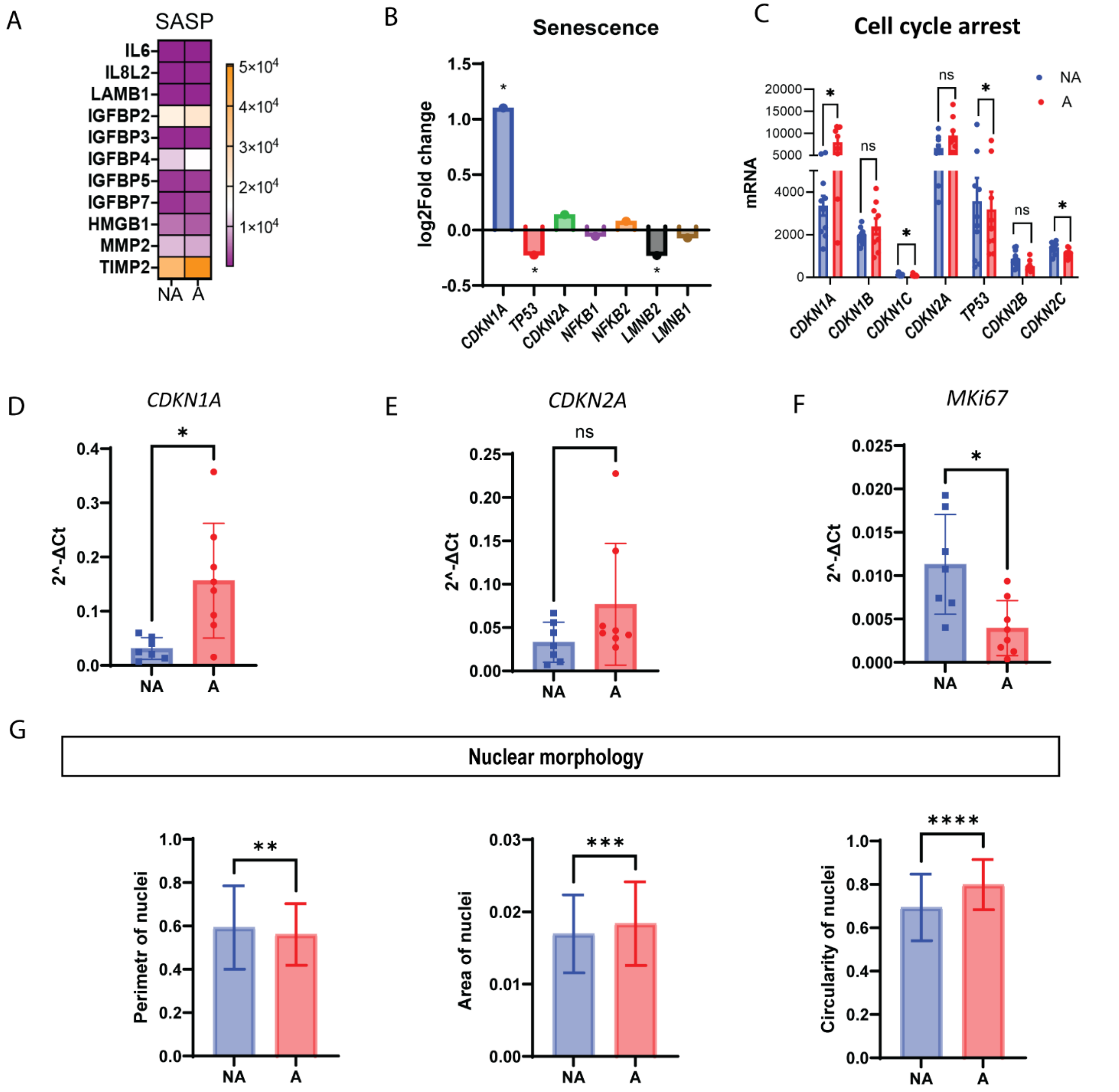

Figure S2.

Senescence and nuclear morphology of MuSCs. (A) SASP genes by RNAseq. **(B)** Gene expression from RNAseq of several senescence markers and **(C)** Cell cycle arrest in MuSCs. RT-qPCR analysis shows **(D)** *CDKN1A* gene expression is significantly increased in affected group and **(E)** *CDKN2A* is not significantly changed between groups. **(F)** *MKi67* significantly decreased in WB affected individuals. The data are presented as the fold change average relative to mean of NA WB, ± SEM. Comparisons between the groups were analyzed using t-test with Brown-Forsythe and Welsh correction (ns $p > 0.05$, * $p \leq 0.05$). **(G)** Nuclear morphology parameters (N=3, in each group) show decrease in perimeter of nuclei, increase in area of nuclei, and increase circularity of affected MuSCs. A, Affected; NA, Non-affected.

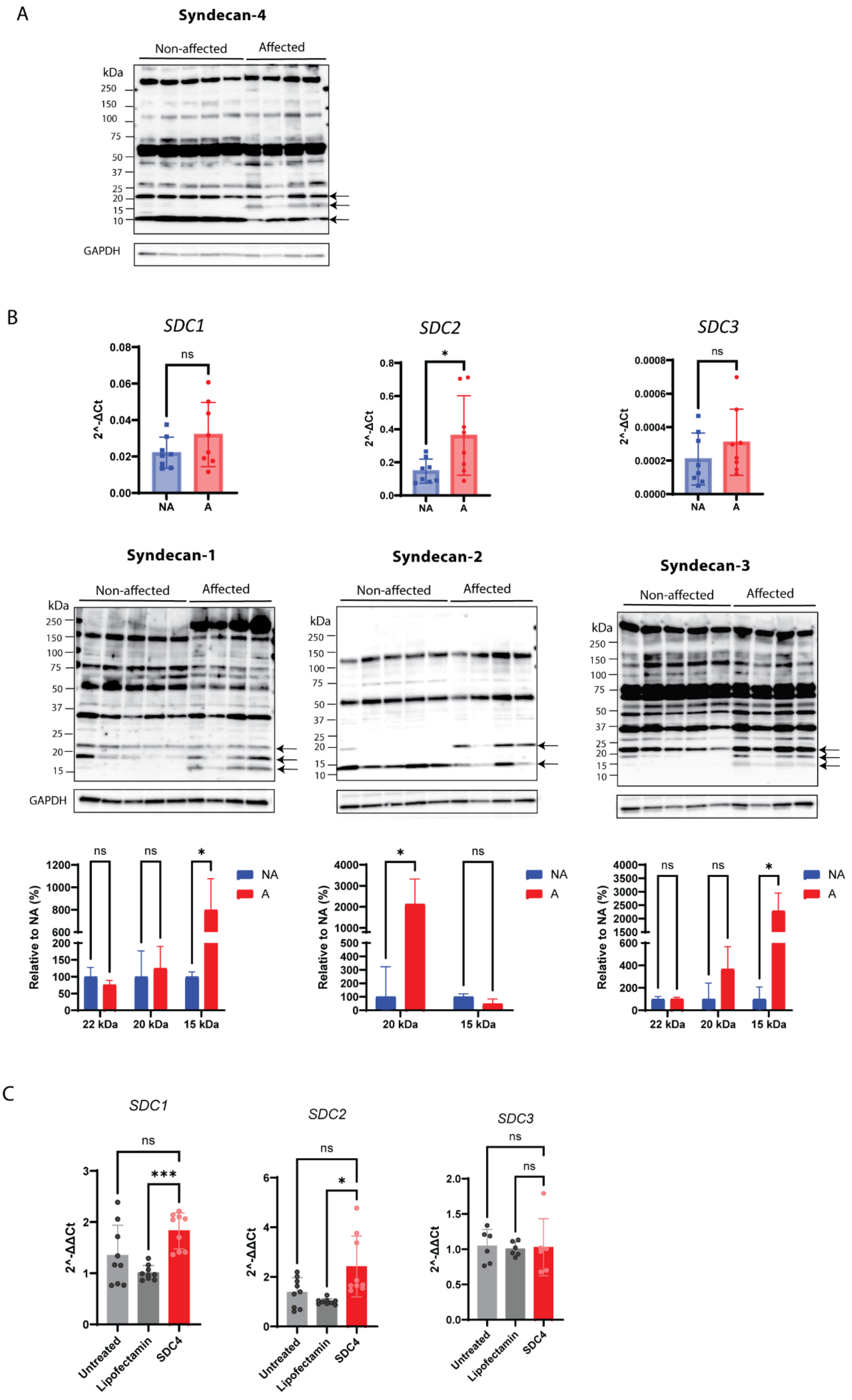

A
Syndecan-4
Non-affected
Affected
kDa
250
150
100
75
50
37
25
20
15
10
GAPDH
B
SDC1
SDC2
SDC3
2^-ΔCt
ns
*
NA
A
Syndecan-1
Syndecan-2
Syndecan-3
Relative to NA (%)
22 kDa
20 kDa
15 kDa
C
2^-ΔΔCt
***
Untreated
Lipofectamin
SDC4

Figure S3.

Gene and protein expression of syndecans *in vitro*. (A) Full length blot of SDC4 protein expression in MuSCs. **(B)** Gene expression and protein and shedding levels of SDC1-3 measured by RT-qPCR and immunoblotting. *SDC2*, but not *SDC1* and *SDC3* was increased in affected animals. The qPCR data are presented as the fold change average relative to mean of mild WB, ± SEM. Significant differences were detected using t-test with Brown-Forsythe and Welsh correction (ns $p > 0.05$, * $p \leq 0.05$). SDC1-3 shedding levels in A (n=4) and NA (n=5) primary MuSCs were analysed by immunoblotting. Quantified bands are highlighted by black arrows (specificity of the bands was confirmed by blocking peptide experiments in Pejšková L., et al, 2023). Increased shedding fragments of 15 kDa, 20 kDa and 15 kDa were detected for SDC1, SDC2 and SDC3 in the affected samples, respectively. Reference protein GAPDH is used as loading control and data are normalized to average of NA group and are presented as a relative percentage to NA. Significant differences were detected using multiple unpair t-test with Brown-Forsythe and Welsh correction (ns $p > 0.05$, * $p \leq 0.05$,). A, Affected; NA, Non-affected. **(C)** Overexpression of SDC4 affected gene expression of SDC1 and SDC2. MuSCs and Lipofectamine were used as controls. Significant differences were detected using One-way ANOVA with Brown-Forsythe and Welsh correction (ns $p > 0.05$, * $p \leq 0.05$, *** $p \leq 0.001$).

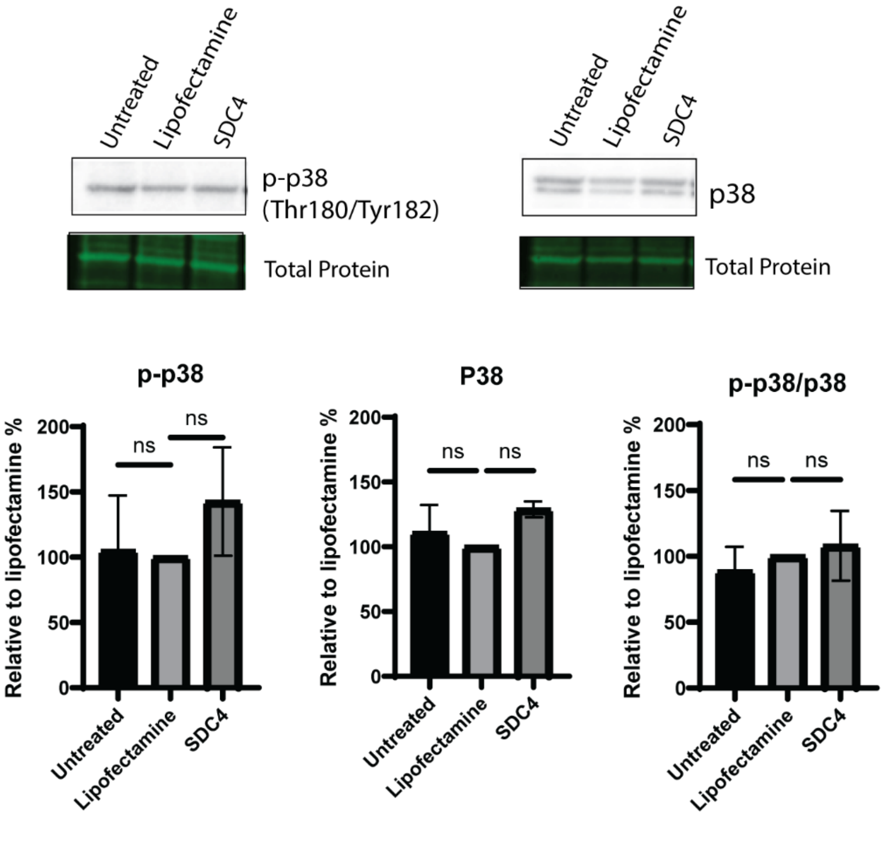

Figure S4.
P38 MAPK signaling in SDC4 overexpressing MuSCs. Protein p38 and phospho(Thr180/Tyr182)-p38 and their ratio show no difference between control (lipofectamine) and SDC4 overexpressed non-affected MuSCs. For control untreated non-affected MuSCs were used. Raw data for each condition (N=3) were normalized to total protein and present it as a relative value to lipofectamine. Significance level was calculated by two-way ANOVA (ns, $p > 0.5$).

Table S1: The list of significantly enriched GO terms for upregulated proteins in WB-affected samples.

source	term_name	term_id	term_size	adjusted_p_value
GO:MF	ATP-dependent protein folding chaperone	GO:0140662	25	9.3890E-14
GO:MF	protein folding chaperone	GO:0044183	39	9.8648E-12
GO:MF	endopeptidase regulator activity	GO:0061135	107	2.2451E-11
GO:MF	peptidase regulator activity	GO:0061134	133	3.3455E-11
GO:MF	unfolded protein binding	GO:0051082	57	3.8296E-10
GO:MF	endopeptidase inhibitor activity	GO:0004866	99	4.5854E-10
GO:MF	peptidase inhibitor activity	GO:0030414	108	2.5256E-09
GO:MF	identical protein binding	GO:0042802	1337	7.7920E-08
GO:MF	enzyme inhibitor activity	GO:0004857	222	8.3253E-08
GO:MF	molecular function inhibitor activity	GO:0140678	267	1.0077E-06
GO:MF	actin binding	GO:0003779	252	1.3150E-06
GO:MF	cytoskeletal protein binding	GO:0008092	615	2.2307E-06
GO:MF	enzyme binding	GO:0019899	1289	8.4794E-06
GO:MF	purine nucleotide binding	GO:0017076	1492	2.3184E-05
GO:MF	ribonucleoside triphosphate phosphatase activity	GO:0017111	476	6.0916E-05
GO:MF	nucleoside phosphate binding	GO:1901265	1610	1.1297E-04
GO:MF	nucleotide binding	GO:0000166	1610	1.1297E-04
GO:MF	cysteine-type endopeptidase inhibitor activity	GO:0004869	13	2.0227E-04
GO:MF	disulfide oxidoreductase activity	GO:0015036	29	2.4704E-04
GO:MF	heterocyclic compound binding	GO:1901363	1705	2.6972E-04
GO:MF	actin filament binding	GO:0051015	107	3.5723E-04
GO:MF	protein binding	GO:0005515	6196	4.1644E-04
GO:MF	carbohydrate derivative binding	GO:0097367	1576	4.3116E-04
GO:MF	pyrophosphatase activity	GO:0016462	519	4.5330E-04
GO:MF	purine ribonucleotide binding	GO:0032555	1408	5.2444E-04

GO:MF	hydrolase activity, acting on acid anhydrides, in phosphorus-containing anhydrides	GO:0016818	523	5.3905E-04
GO:MF	hydrolase activity, acting on acid anhydrides	GO:0016817	524	5.6272E-04
GO:MF	ATP hydrolysis activity	GO:0016887	237	5.6942E-04
GO:MF	ribonucleotide binding	GO:0032553	1423	7.6262E-04
GO:MF	isomerase activity	GO:0016853	115	8.6315E-04
GO:MF	serine-type endopeptidase inhibitor activity	GO:0004867	57	8.9550E-04
GO:MF	protein-disulfide reductase activity	GO:0015035	25	1.1460E-03
GO:MF	intramolecular oxidoreductase activity	GO:0016860	26	1.5326E-03
GO:MF	purine ribonucleoside triphosphate binding	GO:0035639	1370	1.7811E-03
GO:MF	calcium-dependent protein binding	GO:0048306	37	1.8456E-03
GO:MF	anion binding	GO:0043168	1751	2.8838E-03
GO:MF	intramolecular oxidoreductase activity, transposing S-S bonds	GO:0016864	12	3.5794E-03
GO:MF	protein disulfide isomerase activity	GO:0003756	12	3.5794E-03
GO:MF	enzyme regulator activity	GO:0030234	756	4.1633E-03
GO:MF	oxidoreductase activity, acting on a sulfur group of donors	GO:0016667	41	4.1633E-03
GO:MF	adenyl nucleotide binding	GO:0030554	1203	4.3556E-03
GO:MF	ATP-dependent activity	GO:0140657	453	4.5157E-03
GO:MF	phosphoserine residue binding	GO:0050815	7	6.5934E-03
GO:MF	thioredoxin peroxidase activity	GO:0008379	3	7.6219E-03
GO:MF	thioredoxin-dependent peroxiredoxin activity	GO:0140824	3	7.6219E-03
GO:MF	protein kinase C binding	GO:0005080	24	1.2384E-02
GO:MF	small molecule binding	GO:0036094	3566	1.8491E-02
GO:MF	calcium ion binding	GO:0005509	542	2.0102E-02
GO:MF	heat shock protein binding	GO:0031072	81	2.2149E-02
GO:MF	acetyl-CoA C-acyltransferase activity	GO:0003988	4	2.9885E-02
GO:MF	acetyl-CoA C-acetyltransferase activity	GO:0003985	4	2.9885E-02
GO:MF	protein domain specific binding	GO:0019904	385	3.9863E-02

GO:BP	organonitrogen compound metabolic process	GO:1901564	3672	6.6742E-14
GO:BP	protein folding	GO:0006457	119	7.9876E-14
GO:BP	protein maturation	GO:0051604	292	4.7059E-09
GO:BP	protein metabolic process	GO:0019538	2998	6.2521E-09
GO:BP	organonitrogen compound catabolic process	GO:1901565	835	2.1283E-08
GO:BP	chaperone-mediated protein folding	GO:0061077	45	4.1211E-08
GO:BP	protein catabolic process	GO:0030163	590	8.4810E-08
GO:BP	organic substance catabolic process	GO:1901575	1239	4.0759E-07
GO:BP	supramolecular fiber organization	GO:0097435	437	5.2149E-07
GO:BP	macromolecule catabolic process	GO:0009057	832	5.4032E-06
GO:BP	catabolic process	GO:0009056	1502	5.5701E-06
GO:BP	positive regulation of establishment of protein localization to telomere	GO:1904851	8	8.4401E-06
GO:BP	proteolysis involved in protein catabolic process	GO:0051603	442	1.0297E-05
GO:BP	actin cytoskeleton organization	GO:0030036	403	1.5317E-05
GO:BP	regulation of establishment of protein localization to chromosome	GO:0070202	9	2.4813E-05
GO:BP	regulation of establishment of protein localization to telomere	GO:0070203	9	2.4813E-05
GO:BP	small molecule metabolic process	GO:0044281	1028	4.1219E-05
GO:BP	positive regulation of protein localization to chromosome, telomeric region	GO:1904816	10	6.0790E-05
GO:BP	proteolysis	GO:0006508	918	1.2681E-04
GO:BP	regulation of protein localization to chromosome, telomeric region	GO:1904814	12	2.5688E-04
GO:BP	actin filament-based process	GO:0030029	460	3.1746E-04
GO:BP	actin filament organization	GO:0007015	263	5.9145E-04
GO:BP	nucleobase-containing small molecule metabolic process	GO:0055086	379	6.9683E-04
GO:BP	endocytosis	GO:0006897	340	1.0675E-03

GO:BP	positive regulation of telomere maintenance via telomerase	GO:0032212	23	1.2953E-03
GO:BP	establishment of protein localization to telomere	GO:0070200	15	1.3096E-03
GO:BP	carbohydrate metabolic process	GO:0005975	357	2.6204E-03
GO:BP	nucleotide metabolic process	GO:0009117	336	3.0257E-03
GO:BP	ubiquitin-dependent protein catabolic process	GO:0006511	360	3.0505E-03
GO:BP	positive regulation of telomere maintenance via telomere lengthening	GO:1904358	26	3.2681E-03
GO:BP	modification-dependent protein catabolic process	GO:0019941	362	3.3723E-03
GO:BP	protein-containing complex assembly	GO:0065003	966	3.5065E-03
GO:BP	nucleoside phosphate metabolic process	GO:0006753	340	3.7254E-03
GO:BP	modification-dependent macromolecule catabolic process	GO:0043632	365	3.9137E-03
GO:BP	cell-cell recognition	GO:0009988	28	5.6463E-03
GO:BP	pyruvate metabolic process	GO:0006090	81	5.8258E-03
GO:BP	establishment of protein localization	GO:0045184	896	6.1507E-03
GO:BP	binding of sperm to zona pellucida	GO:0007339	19	6.5488E-03
GO:BP	telomere maintenance via telomerase	GO:0007004	40	6.7657E-03
GO:BP	monocarboxylic acid metabolic process	GO:0032787	330	7.5698E-03
GO:BP	phagocytosis	GO:0006909	100	7.8645E-03
GO:BP	ribose phosphate metabolic process	GO:0019693	261	7.9503E-03
GO:BP	purine nucleotide metabolic process	GO:0006163	286	8.9449E-03
GO:BP	establishment of protein localization to chromosome	GO:0070199	20	9.1687E-03
GO:BP	hydrogen peroxide catabolic process	GO:0042744	12	9.2258E-03
GO:BP	glycolytic process through fructose-6-phosphate	GO:0061615	12	9.2258E-03
GO:BP	positive regulation of DNA biosynthetic process	GO:2000573	30	9.3183E-03
GO:BP	positive regulation of organelle organization	GO:0010638	314	1.1317E-02
GO:BP	sperm-egg recognition	GO:0035036	21	1.2580E-02

GO:BP	regulation of vesicle-mediated transport	GO:0060627	270	1.3238E-02
GO:BP	carboxylic acid metabolic process	GO:0019752	495	1.3340E-02
GO:BP	regulation of cellular component organization	GO:0051128	1397	1.3991E-02
GO:BP	regulation of localization	GO:0032879	1110	1.4116E-02
GO:BP	positive regulation of cellular component organization	GO:0051130	635	1.4899E-02
GO:BP	oxoacid metabolic process	GO:0043436	503	1.8122E-02
GO:BP	purine-containing compound metabolic process	GO:0072521	301	1.9689E-02
GO:BP	organic acid metabolic process	GO:0006082	506	2.0288E-02
GO:BP	regulation of endocytosis	GO:0030100	148	2.1332E-02
GO:BP	regulation of protein-containing complex assembly	GO:0043254	233	2.1541E-02
GO:BP	cellular component assembly	GO:0022607	1856	2.2687E-02
GO:BP	regulation of telomere maintenance via telomerase	GO:0032210	34	2.2688E-02
GO:BP	import into cell	GO:0098657	458	2.4983E-02
GO:BP	organophosphate metabolic process	GO:0019637	623	2.5499E-02
GO:BP	cytoskeleton organization	GO:0007010	918	2.6188E-02
GO:BP	proteasomal protein catabolic process	GO:0010498	307	2.6567E-02
GO:BP	regulation of protein metabolic process	GO:0051246	1017	3.1105E-02
GO:BP	purine ribonucleotide metabolic process	GO:0009150	241	3.4079E-02
GO:BP	NADH regeneration	GO:0006735	8	3.6842E-02
GO:BP	regulation of podosome assembly	GO:0071801	8	3.6842E-02
GO:BP	canonical glycolysis	GO:0061621	8	3.6842E-02
GO:BP	glucose catabolic process to pyruvate	GO:0061718	8	3.6842E-02
GO:BP	protein localization to chromosome, telomeric region	GO:0070198	25	3.7881E-02
GO:BP	telomere maintenance via telomere lengthening	GO:0010833	50	3.8397E-02
GO:BP	hexose catabolic process	GO:0019320	16	4.7033E-02
GO:BP	protein stabilization	GO:0050821	119	4.7462E-02

GO:CC	proteasome complex	GO:0000502	46	1.8686E-24
GO:CC	cytosol	GO:0005829	2338	2.5302E-23
GO:CC	endopeptidase complex	GO:1905369	56	8.2333E-22
GO:CC	cytoplasm	GO:0005737	6510	1.2672E-20
GO:CC	peptidase complex	GO:1905368	80	1.7855E-17
GO:CC	proteasome accessory complex	GO:0022624	20	1.3012E-12
GO:CC	proteasome core complex	GO:0005839	14	1.9231E-11
GO:CC	intracellular anatomical structure	GO:0005622	9174	2.7659E-10
GO:CC	intracellular protein-containing complex	GO:0140535	640	4.5170E-10
GO:CC	proteasome regulatory particle	GO:0005838	15	3.7743E-09
GO:CC	sarcomere	GO:0030017	99	9.3338E-09
GO:CC	chaperonin-containing T-complex	GO:0005832	8	1.1229E-08
GO:CC	protein folding chaperone complex	GO:0101031	29	1.1795E-08
GO:CC	myofibril	GO:0030016	114	1.2950E-08
GO:CC	contractile fiber	GO:0043292	118	2.4108E-08
GO:CC	supramolecular fiber	GO:0099512	575	2.6099E-08
GO:CC	supramolecular polymer	GO:0099081	580	3.4386E-08
GO:CC	supramolecular complex	GO:0099080	804	4.9384E-08
GO:CC	Z disc	GO:0030018	59	4.2792E-07
GO:CC	I band	GO:0031674	68	2.7075E-06
GO:CC	proteasome core complex, alpha-subunit complex	GO:0019773	7	4.7487E-05
GO:CC	extracellular space	GO:0005615	647	7.6413E-05
GO:CC	endoplasmic reticulum lumen	GO:0005788	43	2.2890E-04
GO:CC	extracellular region	GO:0005576	951	2.3594E-04
GO:CC	aminoacyl-tRNA synthetase multienzyme complex	GO:0017101	9	2.7332E-04
GO:CC	catalytic complex	GO:1902494	1172	3.9078E-04
GO:CC	cortical cytoskeleton	GO:0030863	58	3.1059E-03
GO:CC	proteasome core complex, beta-subunit complex	GO:0019774	3	3.8241E-03
GO:CC	cell cortex	GO:0005938	164	3.9106E-03

GO:CC	microtubule	GO:0005874	206	1.5433E-02
GO:CC	protein-containing complex	GO:0032991	3938	2.4414E-02
GO:CC	actin cytoskeleton	GO:0015629	311	2.7918E-02
GO:CC	sarcolemma	GO:0042383	62	3.6572E-02
GO:CC	MHC class I peptide loading complex	GO:0042824	5	3.6829E-02
GO:CC	proteasome regulatory particle, lid subcomplex	GO:0008541	5	3.6829E-02
KEGG	Proteasome	KEGG:03050	35	9.2865E-20
KEGG	Glycolysis / Gluconeogenesis	KEGG:00010	49	1.5183E-07
KEGG	Carbon metabolism	KEGG:01200	96	7.4597E-06
KEGG	Protein processing in endoplasmic reticulum	KEGG:04141	144	1.5362E-04
KEGG	Pyruvate metabolism	KEGG:00620	34	2.0210E-03
KEGG	Salmonella infection	KEGG:05132	218	2.0728E-03
KEGG	Pentose phosphate pathway	KEGG:00030	26	2.4506E-03
KEGG	Biosynthesis of amino acids	KEGG:01230	57	1.7746E-02
KEGG	Oocyte meiosis	KEGG:04114	97	2.0225E-02
KEGG	2-Oxocarboxylic acid metabolism	KEGG:01210	26	2.1827E-02
KEGG	Citrate cycle (TCA cycle)	KEGG:00020	26	2.1827E-02
KEGG	Glutathione metabolism	KEGG:00480	47	2.2474E-02
HP	Axial muscle weakness	HP:0003327	38	1.3548E-05
HP	Type 1 muscle fiber predominance	HP:0003803	59	2.0867E-04
HP	Abnormal muscle fiber-type distribution	HP:0033684	74	3.7440E-04
HP	Increased variability in muscle fiber diameter	HP:0003557	106	7.9781E-04
HP	Abnormality of skeletal muscle fiber size	HP:0012084	111	1.4714E-03
HP	Foot dorsiflexor weakness	HP:0009027	121	4.5211E-03
HP	Abnormal circulating creatine kinase concentration	HP:0040081	287	5.8878E-03
HP	Generalized amyloid deposition	HP:0003216	5	7.1087E-03
HP	Abnormality of circulating enzyme level	HP:0011021	291	7.5256E-03
HP	Elevated circulating creatine kinase concentration	HP:0003236	275	9.7426E-03

HP	Muscle fiber inclusion bodies	HP:0100299	36	1.5042E-02
HP	Abnormal muscle fiber morphology	HP:0004303	248	2.1900E-02

Table S2: The list of significantly enriched GO terms for downregulated proteins in WB-affected samples

source	term_name	term_id	adjusted_p_value	term_size
GO:MF	proton-transporting ATP synthase activity, rotational mechanism	GO:0046933	7.4471E-07	12
GO:MF	proton channel activity	GO:0015252	7.2268E-06	20
GO:MF	catalytic activity	GO:0003824	5.9931E-05	4463
GO:MF	proton transmembrane transporter activity	GO:0015078	1.0168E-04	94
GO:MF	ligase activity	GO:0016874	6.6127E-04	137
GO:MF	succinate dehydrogenase (quinone) activity	GO:0008177	2.1382E-03	3
GO:MF	fructose-bisphosphate aldolase activity	GO:0004332	4.2721E-03	4
GO:MF	succinate dehydrogenase activity	GO:0000104	4.2721E-03	4
GO:MF	sugar-phosphatase activity	GO:0050308	1.0659E-02	6
GO:MF	carbohydrate phosphatase activity	GO:0019203	1.4908E-02	7
GO:MF	NAD binding	GO:0051287	1.7623E-02	48
GO:MF	intramolecular phosphotransferase activity	GO:0016868	2.5505E-02	9
GO:MF	aldehyde-lyase activity	GO:0016832	2.5505E-02	9
GO:BP	ATP metabolic process	GO:0046034	6.0162E-11	112
GO:BP	purine ribonucleoside triphosphate metabolic process	GO:0009205	2.3760E-10	130
GO:BP	purine nucleoside triphosphate metabolic process	GO:0009144	3.3595E-10	135
GO:BP	ribonucleoside triphosphate metabolic process	GO:0009199	3.3595E-10	135
GO:BP	small molecule metabolic process	GO:0044281	7.6597E-10	1028
GO:BP	nucleoside triphosphate metabolic process	GO:0009141	7.7924E-10	148

GO:BP	purine ribonucleotide metabolic process	GO:0009150	6.4102E-08	241
GO:BP	ribonucleotide metabolic process	GO:0009259	1.1002E-07	256
GO:BP	ribose phosphate metabolic process	GO:0019693	1.3077E-07	261
GO:BP	proton motive force-driven ATP synthesis	GO:0015986	1.7165E-07	21
GO:BP	generation of precursor metabolites and energy	GO:0006091	2.6070E-07	282
GO:BP	purine nucleotide metabolic process	GO:0006163	2.9551E-07	286
GO:BP	purine-containing compound metabolic process	GO:0072521	4.6542E-07	301
GO:BP	nucleotide metabolic process	GO:0009117	1.2329E-06	336
GO:BP	nucleoside phosphate metabolic process	GO:0006753	1.3687E-06	340
GO:BP	ATP biosynthetic process	GO:0006754	1.4189E-06	31
GO:BP	nucleobase-containing small molecule metabolic process	GO:0055086	3.5621E-06	379
GO:BP	purine ribonucleoside triphosphate biosynthetic process	GO:0009206	4.7692E-06	39
GO:BP	purine nucleoside triphosphate biosynthetic process	GO:0009145	5.4450E-06	40
GO:BP	ribonucleoside triphosphate biosynthetic process	GO:0009201	7.9414E-06	43
GO:BP	nucleoside triphosphate biosynthetic process	GO:0009142	1.4055E-05	48
GO:BP	organophosphate metabolic process	GO:0019637	1.5049E-05	623
GO:BP	carbohydrate derivative metabolic process	GO:1901135	2.6154E-05	660
GO:BP	carboxylic acid metabolic process	GO:0019752	3.6627E-05	495
GO:BP	oxoacid metabolic process	GO:0043436	4.2083E-05	503
GO:BP	organic acid metabolic process	GO:0006082	4.4305E-05	506
GO:BP	cellular respiration	GO:0045333	8.5425E-05	140
GO:BP	energy derivation by oxidation of organic compounds	GO:0015980	5.9154E-04	194

GO:BP	proton motive force-driven mitochondrial ATP synthesis	GO:0042776	7.9297E-04	11
GO:BP	purine ribonucleotide biosynthetic process	GO:0009152	9.8706E-04	111
GO:BP	aerobic respiration	GO:0009060	9.8706E-04	111
GO:BP	carbohydrate metabolic process	GO:0005975	1.1702E-03	357
GO:BP	ribonucleotide biosynthetic process	GO:0009260	1.5156E-03	121
GO:BP	ribose phosphate biosynthetic process	GO:0046390	1.5787E-03	122
GO:BP	purine nucleotide biosynthetic process	GO:0006164	2.0814E-03	129
GO:BP	purine-containing compound biosynthetic process	GO:0072522	2.4207E-03	133
GO:BP	glycolytic process	GO:0006096	3.7230E-03	63
GO:BP	ADP catabolic process	GO:0046032	4.2229E-03	65
GO:BP	pyridine nucleotide catabolic process	GO:0019364	4.2229E-03	65
GO:BP	purine nucleoside diphosphate catabolic process	GO:0009137	4.4907E-03	66
GO:BP	purine ribonucleoside diphosphate catabolic process	GO:0009181	4.4907E-03	66
GO:BP	pyridine-containing compound catabolic process	GO:0072526	4.7709E-03	67
GO:BP	ribonucleoside diphosphate catabolic process	GO:0009191	4.7709E-03	67
GO:BP	ADP metabolic process	GO:0046031	4.7709E-03	67
GO:BP	purine nucleoside diphosphate metabolic process	GO:0009135	5.0638E-03	68
GO:BP	purine ribonucleoside diphosphate metabolic process	GO:0009179	5.0638E-03	68
GO:BP	proton transmembrane transport	GO:1902600	5.3699E-03	69
GO:BP	nucleoside diphosphate catabolic process	GO:0009134	5.6894E-03	70
GO:BP	nucleotide biosynthetic process	GO:0009165	6.5847E-03	163
GO:BP	nucleoside phosphate biosynthetic process	GO:1901293	6.5847E-03	163

GO:BP	ribonucleoside diphosphate metabolic process	GO:0009185	7.1102E-03	74
GO:BP	nucleoside diphosphate metabolic process	GO:0009132	1.0207E-02	81
GO:BP	purine ribonucleotide catabolic process	GO:0009154	1.0207E-02	81
GO:BP	pyruvate metabolic process	GO:0006090	1.0207E-02	81
GO:BP	purine nucleotide catabolic process	GO:0006195	1.2370E-02	85
GO:BP	monocarboxylic acid metabolic process	GO:0032787	1.2953E-02	330
GO:BP	organophosphate biosynthetic process	GO:0090407	1.3409E-02	332
GO:BP	ribonucleotide catabolic process	GO:0009261	1.3571E-02	87
GO:BP	purine-containing compound catabolic process	GO:0072523	1.4856E-02	89
GO:BP	carbohydrate catabolic process	GO:0016052	1.6228E-02	91
GO:BP	nicotinamide nucleotide metabolic process	GO:0046496	2.1774E-02	98
GO:BP	pyridine nucleotide metabolic process	GO:0019362	2.1774E-02	98
GO:BP	nucleotide catabolic process	GO:0009166	2.2668E-02	99
GO:BP	pyridine-containing compound metabolic process	GO:0072524	2.6513E-02	103
GO:BP	nucleoside phosphate catabolic process	GO:1901292	3.0819E-02	107
GO:CC	mitochondrial proton-transporting ATP synthase complex	GO:0005753	4.7077E-10	15
GO:CC	proton-transporting ATP synthase complex	GO:0045259	1.3404E-09	18
GO:CC	proton-transporting ATP synthase complex, catalytic core F(1)	GO:0045261	2.6099E-09	6
GO:CC	proton-transporting two-sector ATPase complex	GO:0016469	1.1540E-07	41
GO:CC	mitochondrial proton-transporting ATP synthase complex, catalytic sector F(1)	GO:0000275	1.7634E-07	3

GO:CC	inner mitochondrial membrane protein complex	GO:0098800	2.3310E-07	108
GO:CC	proton-transporting two-sector ATPase complex, catalytic domain	GO:0033178	2.3596E-07	15
GO:CC	mitochondrial protein-containing complex	GO:0098798	1.1611E-05	207
GO:CC	mitochondrial envelope	GO:0005740	1.3617E-05	369
GO:CC	ATPase complex	GO:1904949	1.4922E-05	106
GO:CC	mitochondrial inner membrane	GO:0005743	1.8059E-05	223
GO:CC	organelle inner membrane	GO:0019866	3.3852E-05	248
GO:CC	mitochondrion	GO:0005739	5.0494E-05	977
GO:CC	mitochondrial membrane	GO:0031966	2.1905E-04	341
GO:CC	organelle envelope	GO:0031967	4.9424E-04	628
GO:CC	envelope	GO:0031975	4.9424E-04	628
GO:CC	catalytic complex	GO:1902494	2.9405E-03	1172
GO:CC	cytoplasm	GO:0005737	9.9291E-03	6510
GO:CC	membrane protein complex	GO:0098796	2.9065E-02	807
KEGG	Metabolic pathways	KEGG:01100	3.3587E-08	1321
KEGG	Oxidative phosphorylation	KEGG:00190	1.2177E-07	99
HP	Skeletal myopathy	HP:0003756	8.6044E-04	8
HP	Infantile onset	HP:0003593	1.0929E-02	1102
